# Comparative Efficacy of Different Repetitive Transcranial Magnetic Stimulation Protocols for Stroke: A Network Meta-Analysis

**DOI:** 10.3389/fneur.2022.918786

**Published:** 2022-06-15

**Authors:** Yuan Xia, Yuxiang Xu, Yongjie Li, Yue Lu, Zhenyu Wang

**Affiliations:** ^1^School of Health Sciences, Wuhan Sports University, Wuhan, China; ^2^School of Life Sciences, Henan University, Kaifeng, China; ^3^Department of Rehabilitation Medicine, Guizhou Provincial Orthopedics Hospital, Guiyang, China

**Keywords:** stroke, motor function, activities of daily living, repetitive transcranial magnetic stimulation, meta-analysis

## Abstract

**Background:**

Although repetitive transcranial magnetic stimulation (rTMS) has been proven to be effective in the upper limb motor function and activities of daily living (ADL), the therapeutic effects of different stimulation protocols have not been effectively compared. To fill this gap, this study carried out the comparison of the upper limb motor function and ADL performance of patients with stroke through a network meta-analysis.

**Methods:**

Randomized controlled trials (RCTs) on the rTMS therapy for stroke were searched from various databases, including PubMed, web of science, Embase, Cochrane Library, ProQuest, Wanfang database, the China National Knowledge Infrastructure (CNKI), and VIP information (www.cqvip.com). The retrieval period was from the establishment of the database to January 2021. Meanwhile, five independent researchers were responsible for the study selection, data extraction, and quality evaluation. The outcome measures included Upper Extremity Fugl-Meyer Assessment (UE-FMA), Wolf Motor Function Test (WMFT), Modified Barthel Index (MBI), the National Institute of Health stroke scale (NIHSS), and adverse reactions. The Gemtc 0.14.3 software based on the Bayesian model framework was used for network meta-analysis, and funnel plots and network diagram plots were conducted using Stata14.0 software.

**Results:**

Ninety-five studies and 5,016 patients were included ultimately. The intervention measures included were as follows: placebo, intermittent theta-burst stimulation (ITBS), continuous theta-burst stimulation (CTBS),1 Hz rTMS,3–5 Hz rTMS, and ≥10 Hz rTMS. The results of the network meta-analysis show that different rTMS protocols were superior to placebo in terms of UE-FMA, NIHSS, and MBI outcomes. In the probability ranking results, ≥10 Hz rTMS ranked first in UE-FMA, WMFT, and MBI. For the NIHSS outcome, the ITBS ranked first and 1 Hz rTMS ranked the second. The subgroup analyses of UE-FMA showed that ≥10 Hz rTMS was the best stimulation protocol for mild stroke, severe stroke, and the convalescent phase, as well as ITBS was for acute and subacute phases. In addition, it was reported in 13 included studies that only a few patients suffered from adverse reactions, such as headache, nausea, and emesis.

**Conclusion:**

Overall, ≥10 Hz rTMS may be the best stimulation protocol for improving the upper limb motor function and ADL performance in patients with stroke. Considering the impact of stroke severity and phase on the upper limb motor function, ≥10 Hz rTMS may be the preferred stimulation protocol for mild stroke, severe stroke, and for the convalescent phase, and ITBS for acute and subacute phases.

**Systematic Review Registration:**

https://www.crd.york.ac.uk/prospero/, identifier [CRD42020212253].

## Introduction

Stroke is a common disease that is detrimental to human health. With high incidence, disability, and recurrence rates, as well as mortality, it puts a heavy financial burden on patients and their families. It has become a leading cause of long-term disability worldwide (1). Post-stroke, patients often experience physical signs, such as postural control abnormalities, impaired balance function, muscular strength, and muscle tension, which will lead to motor dysfunction at different degrees. Studies (2) have proved that about 55–75% of patients with stroke have limited motor function of the upper limb, negatively affecting their activities of daily living (ADL) and quality of life (QOL) (3). Therefore, the difficulty in rehabilitation is based on how to improve the upper limb motor function and ADL performance of patients with stroke effectively and safely.

Based on the interhemispheric competition theory (4, 5), the reciprocal inhibition between these two hemispheres is no longer balanced after a stroke, i.e., the cortical excitability on the affected side is weakened, while that on the unaffected side is strengthened. Hence, the cortex on the contralateral damage side can inhibit the one on the ipsilateral damage side more fiercely, undermining the recovery of motor function in these patients. However, a better overall prognosis can be reached by restoring the balance of this cortical excitability between cerebral hemispheres (6). Therefore, based on this theory, stroke can be treated by inhibiting the excitability on the unaffected side or enhancing it on the affected side.

Repetitive transcranial magnetic stimulation (rTMS) is a neuroelectrophysiological technique that creates an induced electric field in the brain through the time-varying magnetic field of a certain intensity. In this process, neurons are depolarized by the electric field to change the local cortical excitability (7). In accordance with different frequencies, conventional rTMS is classified into low frequency stimulation (≤ 1 Hz, low frequency rTMS, LF-rTMS) and high frequency stimulation (>1 Hz, high-frequency rTMS, HF-rTMS) (8, 9). Theta burst stimulation (TBS) is a new pattern of rTMS, which is divided into intermittent theta-burst stimulation (ITBS) and continuous theta-burst stimulation (CTBS), consisting of low-intensity and short bursts of rTMS at 50 Hz (10). Generally, the LF-rTMS and CTBS can cause a long-term inhibition of synapses by inhibiting the motor cortical excitability, while the HF-rTMS and ITBS will excite the cerebral cortex through its facilitatory effect (11). However, this consensus, which is reached based on physiological effects, is not applicable to all conditions. Therefore, the specific effects are also dependent on the active state of the stimulated site in the brain (12).

Previous meta-analyses (13, 14) have indicated the influence of various stimulation protocols on the upper limb motor function and ADL performance of patients with stroke, but the evidence were obtained by comparison with conventional rehabilitation, while either direct or indirect comparison between different rTMS protocols is missing. Network meta-analysis can provide direct and indirect comparisons among multiple interventions. Hence, we contrast the effects of different rTMS protocols on such function and performance by network meta-analysis so as to provide evidence for the clinical treatment of stroke.

## Materials and Methods

The systematic review in this study was performed as per the Preferred Reporting Items for Systematic Reviews and Meta-Analyses Statement (PRISMA) (15); and this study has been registered in the international prospective register of systematic reviews (PROSPERO), with the registration number of CRD42020212253.

### Inclusion and Exclusion Criteria

The inclusion criteria below were set based on patient, intervention, comparison, outcome, and study design. Inclusion criteria: (1) Patients who met the stroke diagnostic criteria established by WHO (16) or The Fourth National Cerebrovascular Disease Conference in 1995 (17) and were confirmed with this disease through CT or MRI. Moreover, they were in the age range of 18–75 years irrespective of gender and had signed informed consent. (2) In the intervention groups, patients received one of the following treatments: LF-rTMS (1 Hz), HF-rTMS (3, 5, 10, 12, and 20 Hz), ITBS, and CTBS. (3) The placebo comparison included no stimulation and sham stimulation, of which the latter referred to the analog sound without any effective magnetic stimulation. Conventional rehabilitation, such as occupational therapy, physical therapy, virtual reality, and orthosis, was acceptable as cointervention. (4) Primary outcome measures in this study assessed the upper limb motor function and ADL performance, including Upper Extremity Fugl-Meyer Assessment (UE-FMA), Wolf Motor Function Test (WMFT), and Modified Barthel Index (MBI). The UE-FMA and WMFT are commonly used to assess the upper limb motor function in patients with stroke, in the clinic (18, 19). The higher the scores of UE-FMA and WMFT, the better the upper limb motor functions. Moreover, we also assessed the ADL performance through the MBI scale. The MBI scale contains a total of 10 items, with a full score of 100 points (20). A higher MBI score means a better ADL performance. The secondary outcome measures included the National Institute of Health stroke scale (NIHSS) and adverse reactions. The NIHSS scale has a total score of 42 points and is mainly adopted to assess the overall function (21). A higher NIHSS score shows a worse overall function. Meanwhile, adverse reactions were performed to evaluate the security of rTMS.

The studies involving any of the following conditions would be excluded: (1) Patients with upper limb motor dysfunction and ADL impairment were not caused by stroke, but by diseases, such as severe trauma, cerebral palsy, and Parkinson; (2) Quasi-random articles, review studies, case reports; (3) Lack of outcome measures related to the upper limb motor function and ADL performance; (4) Data could not be extracted directly; and (5) Repeatedly published studies.

### Data Sources and Retrieval Strategies

Two reviewers (Y.X and Y.J.L) separately searched for randomized controlled trials (RCTs) about the treatment of stroke by rTMS from PubMed, Embase, Cochrane Library, Web of Science, ProQuest, CNKI, Wanfang database, and VIP information. The retrieval period starts from the establishment of the database to January 2021. Taking PubMed as an example, the retrieval strategies are shown below: (Stroke[Mesh]OR cerebrovascular accident[Title/Abstract]OR CVA[Title/Abstract] OR Brain Vascular Accident[Title/Abstract] OR hemiplegia[Title/Abstract] OR (apoplexy[Title/Abstract] OR (hemiparesis[Title/Abstract]) AND (repetitive transcranial magnetic stimulation[Title/Abstract] OR Transcranial Magnetic Stimulation[Title/Abstract]OR TMS[Title/Abstract] OR rTMS[Title/Abstract]OR Theta burst stimulation[Title/Abstract] OR θ burst stimulation[Title/Abstract]). These search terms and expressions with the same meanings were also retrieved in Chinese databases.

### Study Selection and Data Collection

After the duplicated data were eliminated by EndnoteX9, two reviewers (Y.X and Y.X.X) filtered the references by reading titles and abstracts. Then, they browsed the entire article to further select those that could meet the inclusion criteria, and the disagreements arising from this process were settled through group discussion or by the reviewer with rich consulting experience. All excluded studies and related reasons were recorded.

The following data of references were extracted from each study by two reviewers (Z.Y.W and Y.L) using preassigned tables: the first author, year and country of publication, sample size, age, interventions, parameters of stimulation (frequency and number of pulses), course of disease, stimulation site, and outcome measures. Then, the collected data were put into Excel sheets to be cross-checked by two reviewers. Any disagreement should be solved through discussion or by Li.

### Risk of Bias

Cochrane Collaboration's tool (22) for assessing the risk of bias was used by two reviewers (Y.J.L and Y.X) separately to evaluate the risk of bias in the included studies according to the contents below: generation of random sequences, allocation concealment, blinding of subjects and therapists, blind outcome assessment, incomplete outcome data, selective reporting results, and other biases. Then, the results were reviewed by three reviewers (Y.X.X, Y.L, and Z.Y.W), who assessed these studies to be low, high, or unclear risk of bias based on the *Cochrane Handbook for Systematic Reviews of Interventions*. In case of any disagreement, the research group would settle it through discussion.

### Statistical Analysis

#### Network Meta-Analysis

In this study, the Stata14.0 software was used to draw the network diagram for comparing various therapies. Since the outcome indicators herein were continuous variables and were assessed by the same scale, the weighted mean difference (WMD) and 95% confidence interval (CI) were taken as the effect size. In addition, we conducted the network meta-analysis using the GeMTC 0.14.3 software based on the Bayesian framework. The specific parameter details were as follows: the initial value was set to 2.5; four chains were built by Markov Chain Monte Carlo (MCMC) method for the simulation that was iterated 50,000 times, of which the first 20,000 ones were used for annealing (23). Meanwhile, the node-splitting method was applied to verify the inconsistency between direct and indirect evidence, with *P* > 0.05 indicating the non-significance of this inconsistency. The convergence of the included studies was represented by the potential scale reduced factor (PSRF), and the value that was close to or equal to 1 indicated a good convergence. Then, the consistency model was analyzed to obtain high-reliability results (24).

The therapeutic effects of different interventions were sequenced by drawing the figure of ranking probability, with the probability of each grade ranging between 0 and 100%. ForUE-FMA, WMFT, and MBI, in which higher score meant better function, the higher the probability of the first grade, the more probable the best intervention effects. Conversely, the intervention was more likely to be optimal in the case of a higher probability of the last grade, which was applicable to the NIHSS, an indicator with a lower score representing better function.

#### Subgroup and Sensitivity Analyses

We carried out subgroup and sensitivity analyses to verify the robustness of our results. Subgroup analyses were performed according to phase of stroke and degree of stroke severity and sensitivity analyses were carried out by removing the studies with a sample size of <10.

#### Publication Bias

We evaluated the publication bias of these studies by visually measuring the symmetry of funnel plots in Stata14.0 software.

## Results

### Search Results and Study Characteristics

**Figure 1:**
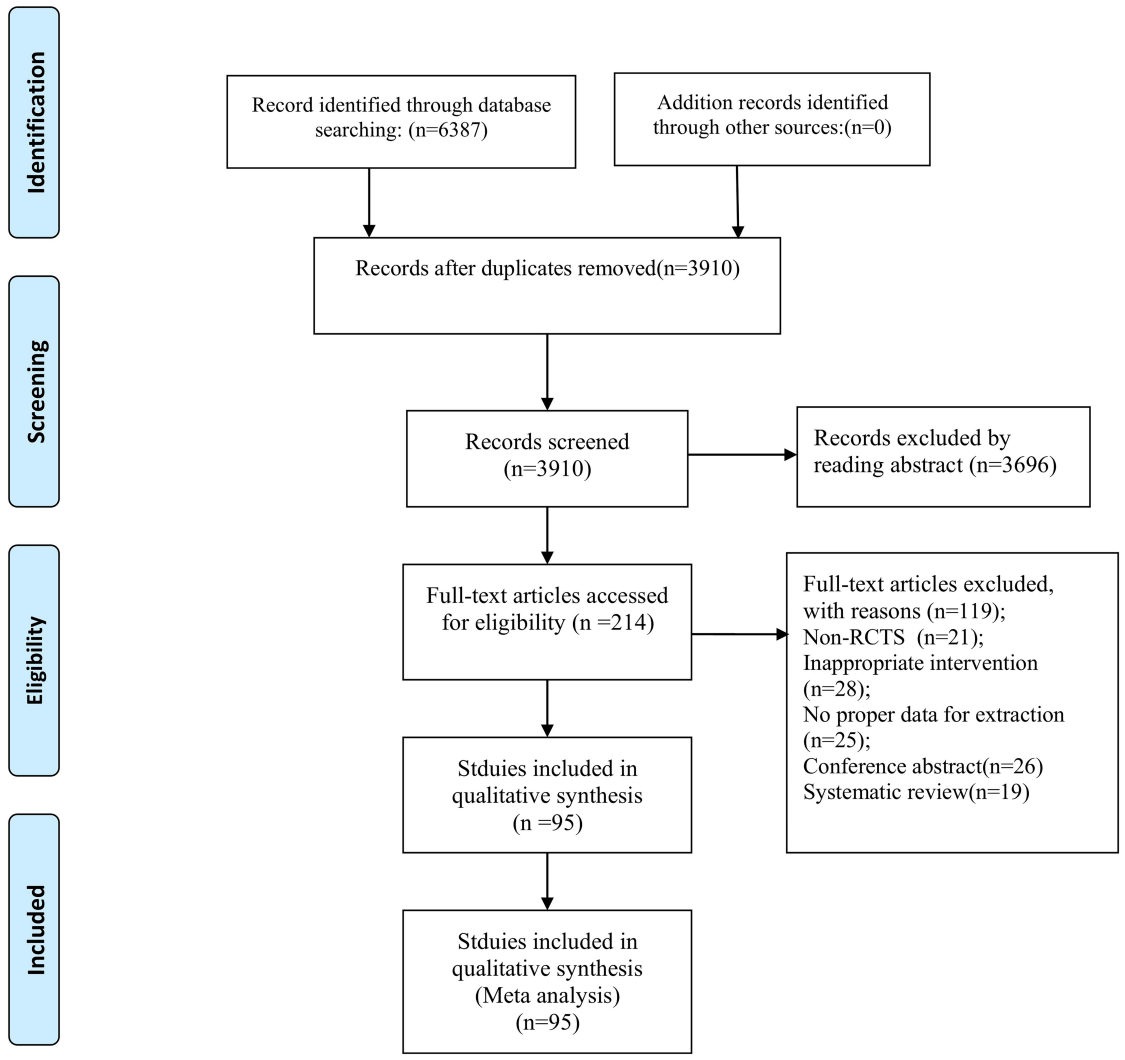
Flowchart of the study selection process.

A flowchart of the study selection process is shown in Figure 1. A total of 6,387 studies were retrieved. Thereinto, we read the full article from 214 studies, of which 119 studies were excluded as they failed to meet the preassigned inclusion criteria. Ultimately, 95 RCTs, involving 5,016 patients, were qualified for the meta-analysis. Among these trials, there were 11 studies related to ITBS, 3 studies related to CTBS, 55 studies related to LF-rTMS, and 26 studies related to HF-rTMS. Supplementary Table 1 presents the basic characteristics of all included studies.

### Quality Evaluation

Supplementary Table 2 shows the results of the quality evaluation of the included studies. Thereinto, 59 studies reported the specific random mode; 24 revealed the detailed allocation concealment; 51 applied the double-blind or single-blind trials; 13 studies showed a small probability of selection bias; and only 1 study did not explain the concrete causes of missing data.

### Network Meta-Analysis

#### Upper Extremity Fugl-Meyer Assessment

**Figure 2:**
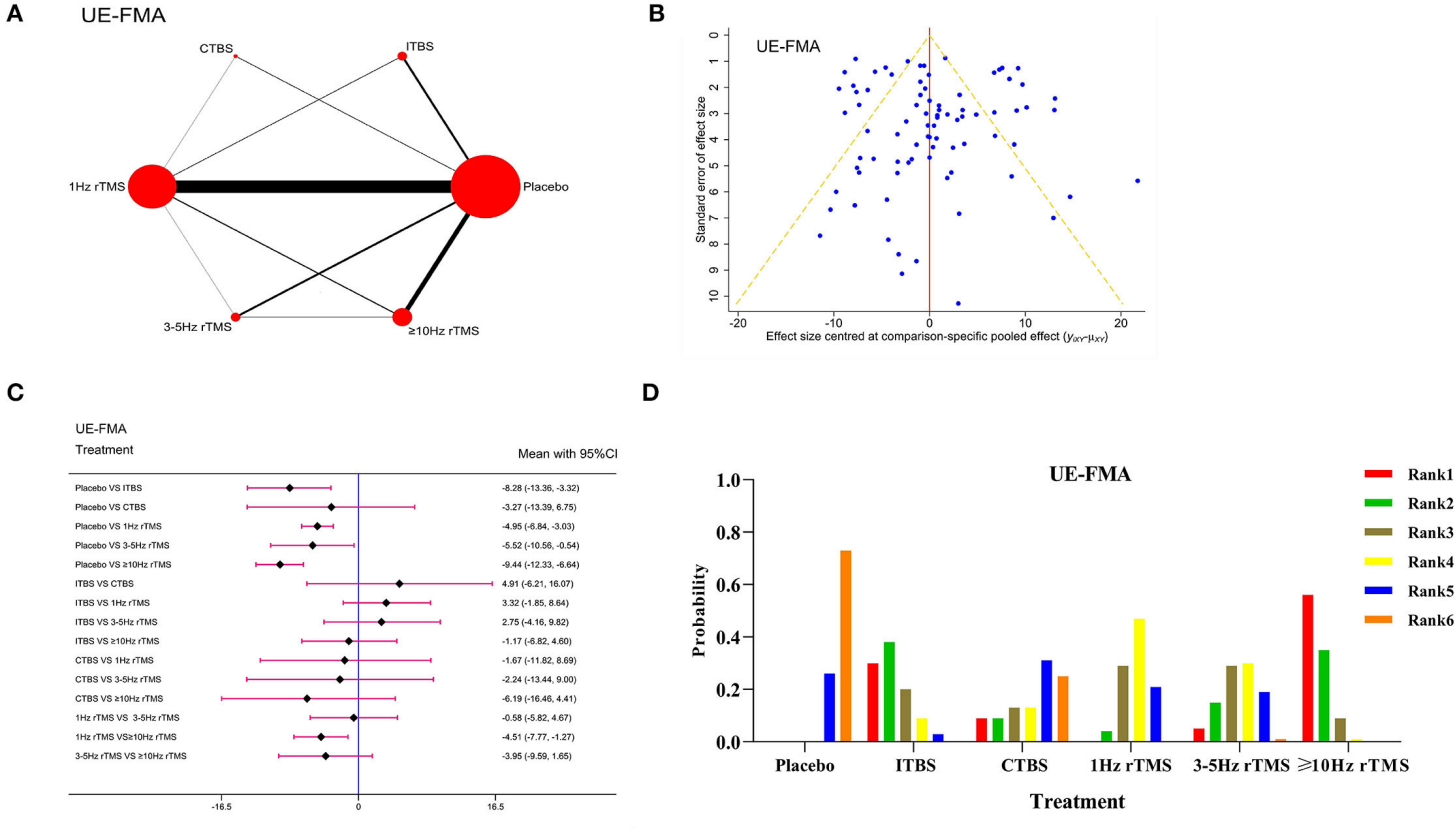
Network meta-analysis results for Upper Extremity Fugl-Meyer Assessment (UE-FMA). **(A)**: Network plot; **(B)**: Funnel plot; **(C)**: Forest plot; **(D)**: The figure of ranking probability.

Sixty-eight studies and 3,470 patients were included. The network evidence diagram is presented in Figure 2A.The PSRF value was 1, indicating satisfactory convergence (Supplementary Table 3). According to Supplementary Table 4, the good consistency could be verified by the node-splitting method. The funnel plot was basically symmetrical, as shown in Figure 2B. Besides, the network meta-analysis results in the forest plot are shown in Figure 2C. Compared with the placebo, ITBS [WMD = −8.28, 95% CI (−13.36, −3.32)], 1 Hz rTMS [WMD = −4.95, 95% CI (−6.84, −3.03)] and ≥10 Hz rTMS [WMD = −9.44, 95%CI (−12.33, −6.64)] had better curative effects. Also, ≥10 Hz rTMS on the ipsilateral damage side had better curative effects than 1 Hz rTMS on the contralateral damage side [WMD = −4.51, 95% CI(−7.77, −1.27)]. In the meantime, the ranking probability of grades (Figure 2D) proved that the ≥10 Hz rTMS was the best protocol to enhance the UE-FMA score. Here, we also performed the sensitivity analysis by removing the studies with a sample size of <10 to verify the robustness of our results. Since network meta-analysis results in forest plot (Supplementary Figure 1A), as well as the ranking probability of grades (Supplementary Figure 1B), were not significantly different from the overall results, we verified the stability and reliability of our results.

#### Wolf Motor Function Test

**Figure 3:**
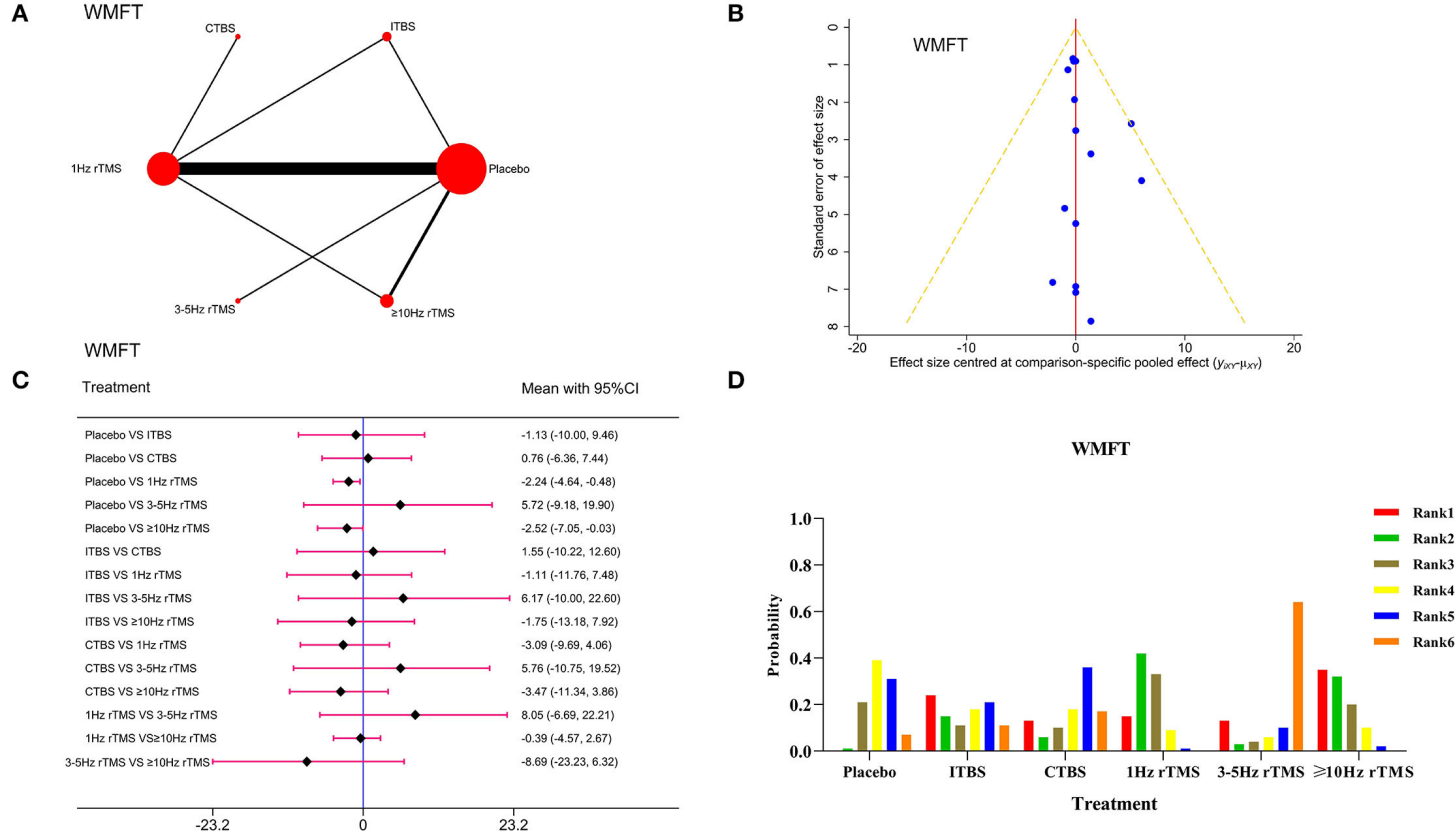
Network meta-analysis results for Wolf Motor Function Test (WMFT). **(A)**: Network plot; **(B)**: Funnel plot; **(C)**: Forest plot; **(D)**: The figure of ranking probability.

There were 11 studies and 772 patients with respect to WMFT. Figure 3A shows the network evidence diagram. The PSRF value moved close to 1, indicating satisfactory convergence (Supplementary Table 3). The good consistency could be verified by the node-splitting method (*P* > 0.05), as presented in Supplementary Table 4. The funnel plot in Figure 3B was almost symmetrical. Forest plot in Figure 3C showed that compared with the placebo, 1Hz rTMS [WMD = −2.24, 95% CI (−4.64, −0.48)] and ≥10 Hz rTMS [WMD = −2.52, 95% CI (−7.05, −0.03)] were significantly more effective. However, there was no statistical difference between the different rTMS protocols. In addition, according to the ranking probability (Figure 3D), the ≥10 Hz rTMS was the best protocol.

#### Modified Barthel Index

**Figure 4:**
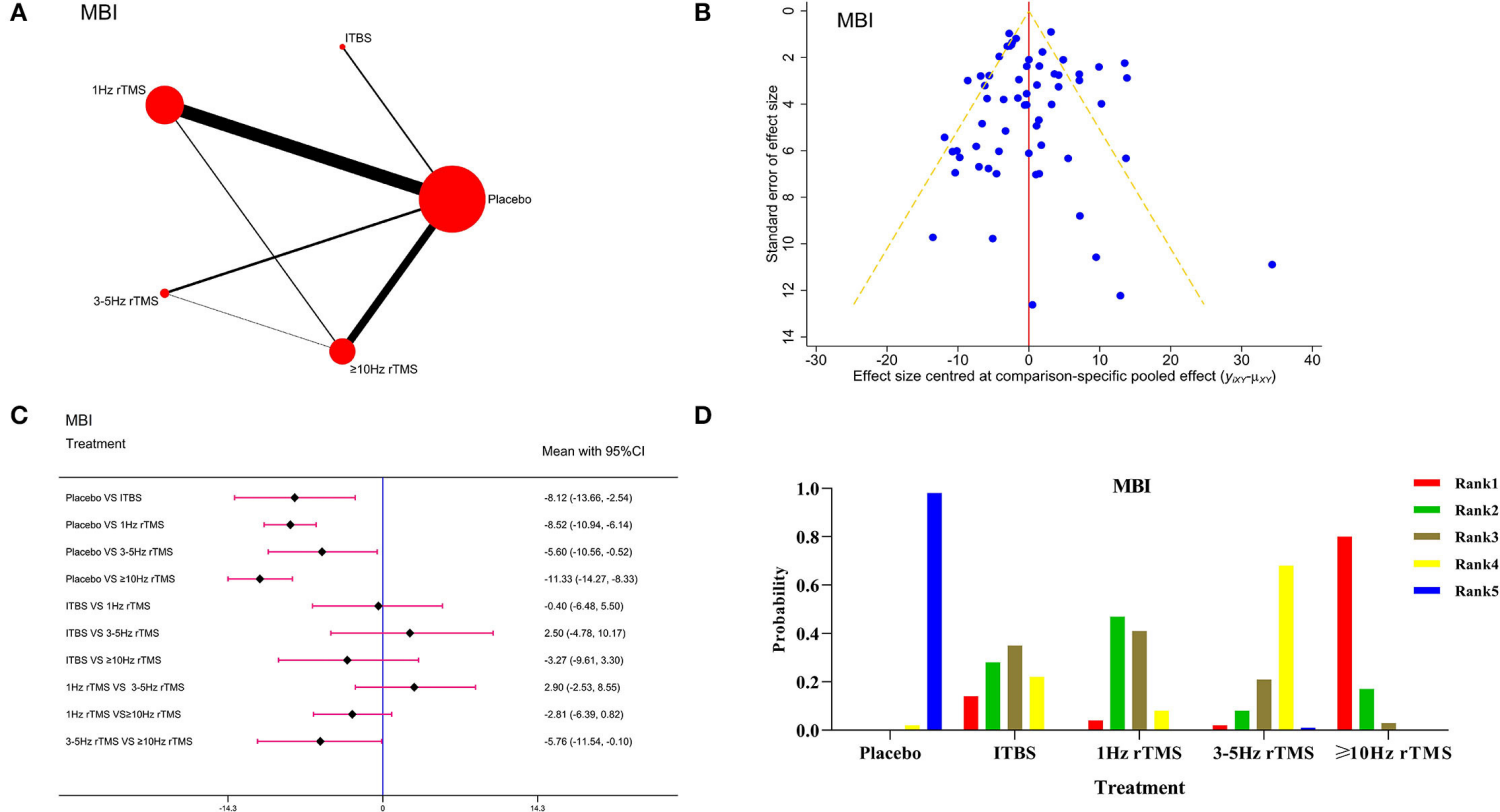
Network meta-analysis results for Modified Barthel Index (MBI). **(A)**: Network plot; **(B)**: Funnel plot; **(C)**: Forest plot; **(D)**: The figure of ranking probability.

Fifty-five studies involving 2,862 patients were included. The network evidence diagram is presented in Figure 4A.The PSRF value was 1, indicating satisfactory convergence (Supplementary Table 3). As shown in Supplementary Table 4, the good consistency could be verified by the node-splitting method (*P* > 0.05), and funnel plot was almost symmetrical (Figure 4B). According to the Forest plot results in Figure 4C, ITBS [WMD = −8.12, 95% CI (−13.66, −2.54)], 1Hz rTMS [WMD = −8.52, 95% CI (−10.94, −6.14)], 3–5 Hz rTMS [WMD = −5.60, 95% CI (−10.56, −0.52)] and ≥10 Hz rTMS [WMD = −11.33, 95% CI (−14.27, −8.33)] were significantly more effective than placebo. Also, ≥10 Hz rTMS on the ipsilateral damage side had better curative effects than 3–5 Hz rTMS on the ipsilateral damage side [WMD = −5.76, 95% CI (−11.54, −0.10)].The picture of ranking probability in Figure 4D shows that the rTMS at ≥10 Hz could enhance the MBI score effectively and was considered as the most effective protocol.

Meanwhile, to verify the robustness of our results, we excluded the studies with a sample size of <10 for the sensitivity analysis and found that network meta-analysis results (Supplementary Figure 2A) and probability ranking of grades (Supplementary Figure 2B) were in line with the overall results, indicating the stability and reliability of results.

#### The National Institute of Health Stroke Scale

**Figure 5:**
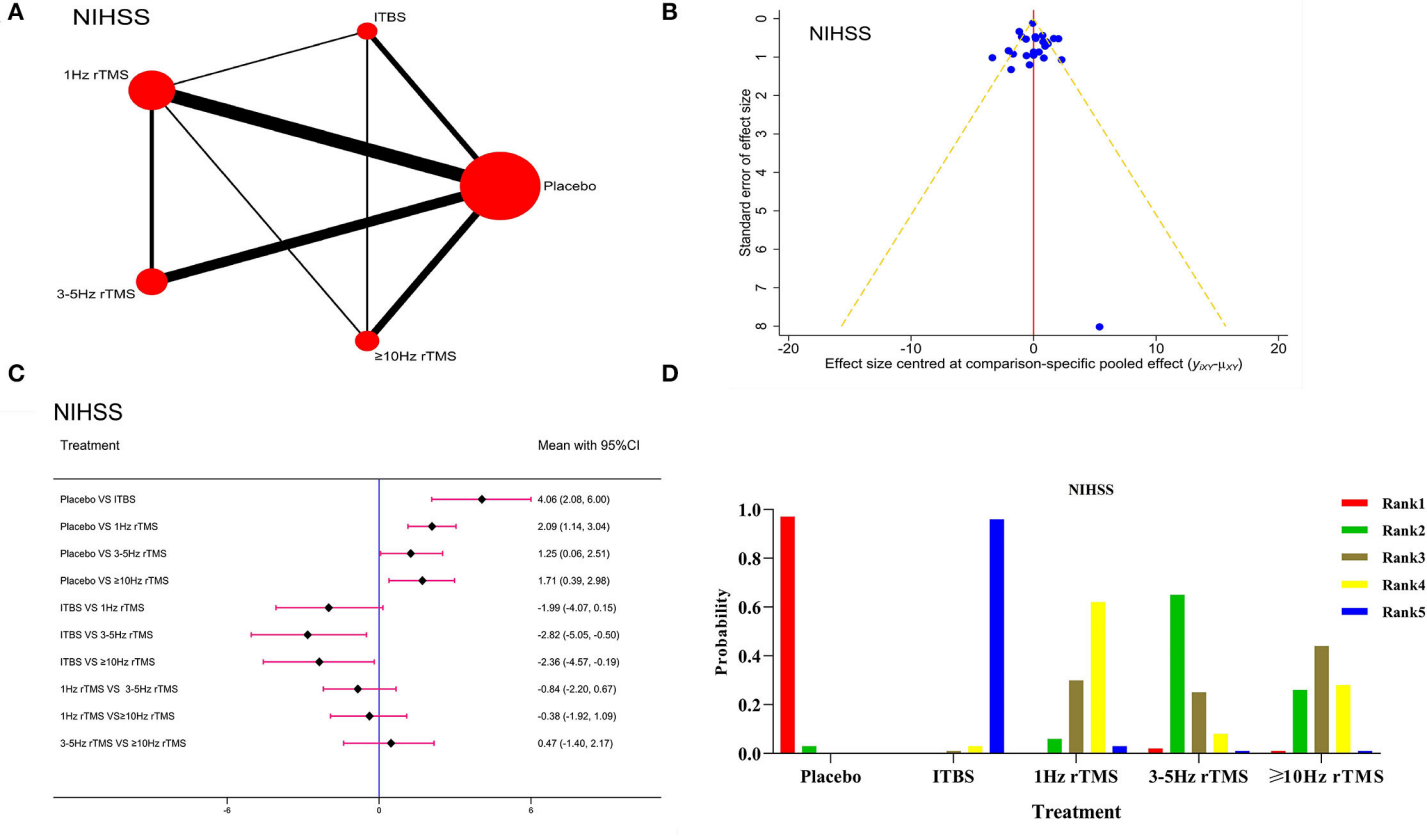
Network meta-analysis results for the National Institute of Health stroke scale (NIHSS). **(A)**: Network plot; **(B)**: Funnel plot; **(C)**: Forest plot; **(D)**: The figure of ranking probability.

Seventeen studies and 841 patients were included. Figure 5A shows the network evidence diagram. The PSRF value was 1, indicating satisfactory convergence (Supplementary Table 3). The consistency was proved to be good by the node-splitting method (*P* > 0.05) in Supplementary Table 4. The funnel plot (Figure 5B) was basically symmetrical. Forest plot in Figure 5C showed that, compared with the placebo, ITBS [WMD = 4.06, 95% CI (2.08, 6.00)], 1 Hz rTMS [WMD = 2.09, 95% CI (1.14, 3.04)], 3–5 Hz rTMS [WMD = 1.25, 95% CI (0.06, 2.51)] and ≥10 Hz rTMS [WMD = 1.71, 95% CI (0.39, 2.98)] had better curative effects. Also, ITBS on the ipsilateral damage side had better curative effects than 3–5 Hz rTMS [WMD = −2.82, 95% CI (−5.05, −0.50)] and ≥10 Hz rTMS [WMD = −2.36, 95% CI (−4.57, −0.19)] on the ipsilateral damage side. Among them, the ITBS was the optimal one in this aspect, as shown by the ranking probability in Figure 5D. Besides, we carried out the sensitivity analysis by excluding the studies with a sample size of <10 to verify the robustness of our results, which were stable and reliable because there was no obvious change in the network meta-analysis results (Supplementary Figure 3A) and ranking probability (Supplementary Figure 3B).

### Subgroup Analyses

In our opinion, different levels of stroke severity may cause rTMS to have different therapeutic effectiveness. A previous study (25) has used half of the UE-FMA score as the boundary between mild and severe stroke. For this reason, we set two subgroups for UE-FMA: mild stroke group (UE-FMA score ≥33) and severe stroke group (UE-FMA score <33). In the mild stroke group (UE-FMA score ≥33), network meta-analysis results in the forest plot (Supplementary Figure 4A) demonstrated that compared with the placebo, 1 Hz rTMS and ≥10 Hz rTMS had better curative effects, with a statistically significant difference (*P* < 0.05). There was no statistical difference between the different rTMS protocols. According to the ranking probability in Supplementary Figure 4B, the ≥10 Hz rTMS was the best protocol. In the severe stroke group (UE-FMA score <33), network meta-analysis results in the forest plot (Supplementary Figure 5A) demonstrated that compared with the placebo, ITBS, 1 Hz rTMS,3–5 Hz rTMS, and ≥10 Hz rTMS had better curative effects, with a statistically significant difference (*P* < 0.05). Also, ≥10 Hz rTMS on the ipsilateral damage side had better curative effects than 1 Hz rTMS on the contralateral damage side. According to the ranking probability in Supplementary Figure 5B, the ≥10 Hz rTMS was the best protocol.

Moreover, we performed a subgroup analysis for UE-FMA according to the phase of stroke, including the acute phase and subacute phase group (<1 month) and convalescent phase group (>1 month) (26, 27). In the acute phase and the subacute phase groups, network meta-analysis results in forest plot (Supplementary Figure 6A) demonstrated that compared with the placebo, the ITBS,1 Hz rTMS and ≥10 Hz rTMS had better curative effects, with a statistically significant difference (*P* < 0.05). The ITBS on the ipsilateral damage side had better curative effects than 3-5Hz rTMS on the ipsilateral damage side (*P* < 0.05). According to the ranking probability in Supplementary Figure 6B, the ITBS was the best protocol. In the convalescent group, forest plot in Supplementary Figure 7A shows that ≥10 Hz rTMS had better curative effects than placebo,1 Hz rTMS and ITBS (*P* < 0.05). Among them, the ≥10 Hz rTMS was the optimal one in this aspect, as shown by the ranking probability in Supplementary Figure 7B.

### Adverse Reactions

Adverse reactions were reported in 13 of the 95 included studies (Supplementary Table 5). Among the 3 cases of epilepsy identified, 2 cases had epileptic seizures after the first treatment, and the reason was that the researcher did not fully notice the epileptic signs during the initial screening; the other 1 case developed epilepsy symptoms after the fourth treatment, which may be related to phenytoin treatment. Also, there were 15 cases of mild headache, 3 cases of dizziness, and 2 cases of scalp discomfort, all of which disappeared after the treatment. In addition, a study noted that a small number of patients experienced numbness in the facial muscles during treatment, and the numbness disappeared after treatment.

## Discussion

This study provides evidence for the recovery of the upper limb motor function and ADL performance in patients with stroke who were treated with rTMS. The network meta-analysis showed that the TBS, LF-rTMS, and HF-rTMS could improve the upper limb motor function more effectively than the placebo; and among them, the rTMS at ≥10 Hz achieved the best efficacy in this regard. Moreover, the ADL performance of the stimulation groups was improved more obviously than that of the placebo group; and the ≥10 Hz rTMS may be the optimal protocol to promote the recovery of patients with stroke. The subgroup analysis of UE-FMA confirmed that ≥10 Hz rTMS had a greater efficacy in patients with mild and severe strokes and in the convalescent phase, and ITBS provided more benefits during the acute and subacute phases. Meanwhile, by summarizing the adverse reactions mentioned in all included studies, we found that only a few patients suffered from mild headache and dizziness. Therefore, the rTMS, with high safety, deserves to be generalized in the clinical practice.

Post-stroke, patients often experience multiple dysfunctions, mostly in upper limbs, which will seriously affect their QOL (28). Besides, compared with lower extremities, the upper ones are recovered relatively slowly, with unsatisfactory rehabilitation effects. Hence, the rehabilitation of these patients mainly lies in the recovery of upper extremities (29). UE-FMA and WMFT, with good reliability and validity, have been widely used to assess the upper limb motor function (30). Therefore, we also adopted these two scales to evaluate such functions in patients with stroke and finally found that the HF-rTMS at ≥10 Hz and the LF-rTMS could achieve better effects in this respect than placebo. Meanwhile, the HF-rTMS at ≥10 Hz showed better efficacy than the LF-rTMS. However, our findings were slightly different from the results of previous meta-analysis on RCTs (31). For the consistent part, both analyses indicated that the motor function of patients with stroke could be improved by HF-rTMS and LF-rTMS; but contrary to our results, Hsu et al. (32) believed that the LF-rTMS on the unaffected side was more effective than the HF-rTMS on the affected side. The difference between these two analyses was that we integrated the direct and indirect comparisons and included more studies to expand the sample size. Besides, in our view, the motor function of patients after a severe stroke could not be recovered effectively by correcting the imbalance between hemispheres through the inhibitory effect of LF-rTMS on the excitability of the unaffected side. This is because the inhibition is weak across the corpus callosum when the cerebral hemispheres are seriously injured, causing the residual neurons on the affected side to be unable to supplement the lost function; and accordingly, the functional recovery of patients with stroke is more dependent on the hemisphere of the unaffected side (33–35). Thus, inhibiting this side may interfere with the motor function recovery on the affected side (36).

Furthermore, Takeuchi et al. (37) also indicated that the low-frequency stimulation on the unaffected side of patients with stroke affected the coordination of their hands. Meanwhile, by building the mouse models with stroke, Gao et al. (38) et al. found that the HF-rTMS inhibited the neuronal apoptosis and glucose maintenance in the diseased hemisphere. Besides, considering the effects of the disease course of the patients on the efficacy of rTMS at different protocols, we also carried out the subgroup analysis. A previous study (25) has used half of the UE-FMA score as the boundary between mild and severe stroke. In order to validate the above conjectures, we classified the patients with more than 33 UE-FMA score as those with severe stroke and found that the HF-rTMS at ≥10 Hz was superior to other stimulation protocols in improving the upper limb movements of patients with mild and severe stroke. At the same time, we performed the sensitivity analysis by excluding the studies whose sample size of a single group was <10, and the results also proved that the HF-rTMS at ≥10 Hz was the optimal one. Therefore, based on existing evidence, the upper limb motor function of patients with stroke can be positively affected by different stimulation protocols, of which the ≥10 Hz HF-rTMS is the best intervention. In addition, we also performed subgroup analysis according to the phase of stroke. The results showed that in the acute phase and in the subacute phase groups, ITBS was the optimal stimulation protocol in improving the upper limb motor function in patients with stroke. However, in the convalescent phase group, ≥10 Hz rTMS was the best protocol which was superior to ITBS. Previous studies (39, 40) indicated that HF-rTMS enhanced only the cortical excitability of the affected M1. In contrast, ITBS could modulate interhemispheric imbalance by increasing the cortical excitability of the affected M1 and suppressing that of the unaffected M1. It is also worth noting that compared with traditional HF-rTMS, ITBS is closer to the natural neuron firing protocol of the brain, which can better regulate the cortical environment, thereby improving the motor function (41, 42). Therefore, in the case of severe excitatory imbalance between the two hemispheres in the acute phase of stroke, ITBS can adjust the excitatory(E)/inhibitory(I) balance to a more optimal ratio. In the convalescent phase, the excitability of the bilateral hemispheres of the brain tends to be balanced, and ITBS may reduce the compensatory effect of the contralateral hemisphere by inhibiting the excitability of the contralateral hemisphere (33), thereby hindering the functional recovery after stroke, so its efficacy may be worse than ≥10 Hz HF-rTMS. Chou et al. (43) also recommended the application of HF-rTMS in patients with stroke patients during the convalescent phase, considering its mechanism of action and potential adverse effects.

Usually, post-stroke, patients have motor dysfunction of varying degrees, affecting their ADL performance frequently. MBI is the most widely used method to evaluate this performance, with good reliability, validity, and sensitivity (44). Results showed that the ITBS, as well as the rTMS at 1 Hz, 3–5Hz and ≥10 Hz, could enhance the MBI score more effectively than the placebo. In the meantime, the ranking probability results also indicated that the ≥10 HzrTMS was the optimal protocol to improve the MBI of patients with stroke, which was consistent with the UE-FMA. Branco et al. (45) and Fujita et al. (46) pointed out that the upper limb motor function had an obvious positive correlation with the ADL performance. Therefore, the HF-rTMS at ≥10 Hz could further promote the recovery of this performance by improving these motor functions.

The NIHSS showed good reliability and validity in evaluating the stroke severity (47). According to the ranking probability in our results, the ITBS had the best effect on relieving this severity. However, there was no statistical difference among intervention groups, and only a few studies were included, meaning that the ranking results should be treated critically. Besides, to ensure the objectivity of research results, more high-quality RCTs with larger sample sizes are needed for further verification.

## Limitations

Although we included all intervention measures in the network meta-analysis to obtain comprehensive results, our study had certain limitations. First, the intervention duration, stimulated parts, frequency, and pulse were not precisely identical in the included studies, resulting in potential heterogeneity. Second, despite the inclusion of complete stimulation protocols in this analysis, the intervention data about the rTMS at 1 Hz and ≥10 Hz accounted for 57.89 and 27.37%. In comparison, those of ITBS and CTBS merely contributed to 11.57 and 3.16% of the total data, respectively. The shortage of direct evidence probably undermined the validity. Therefore, the results should be treated with caution. Third, since the references were either in English or Chinese, there may be a problem of language bias.

## Conclusion

Overall, ≥10 Hz rTMS may be the greatest stimulation protocol for improving the upper limb motor function and ADL performance of patients with stroke. Considering the impact of stroke severity and phase on upper limb motor function, ≥10 Hz rTMS may be the preferred stimulation protocol for mild and severe strokes and in the phase of convalescent and ITBS for acute and subacute phases. However, due to research limitations, the conclusion drawn by this study needs to be further verified by more high-quality studies.

## Data Availability Statement

The original contributions presented in the study are included in the article/Supplementary Material, further inquiries can be directed to the corresponding author.

## Author Contributions

YLi, YXi, and YXu drafted the manuscript. ZW and YLu reviewed the manuscript. All authors participated in conducting the article retrieval, selection of studies, data extraction, and assessment of risk of bias. Moreover, final approval was provided by all the authors.

## Conflict of Interest

The authors declare that the research was conducted in the absence of any commercial or financial relationships that could be construed as a potential conflict of interest.

## Publisher's Note

All claims expressed in this article are solely those of the authors and do not necessarily represent those of their affiliated organizations, or those of the publisher, the editors and the reviewers. Any product that may be evaluated in this article, or claim that may be made by its manufacturer, is not guaranteed or endorsed by the publisher.

## References

[1] Benjamin EJ Virani SS Callaway CW Chamberlain AM Chang AR Cheng S . Heart Disease and Stroke Statistics-2018 Update: a report from the American Heart Association. Circulation. (2018) 137:e67–67e492. 10.1161/CIR.0000000000000558 29386200

[2] Wolf SL Winstein CJ Miller JP Taub E Uswatte G Morris D . Effect of constraint-induced movement therapy on upper extremity function 3 to 9 months after stroke: the EXCITE randomized clinical trial. JAMA. (2006) 296:2095–104. 10.1001/jama.296.17.2095 17077374

[3] Carlsson H Gard G Brogårdh C. Upper-limb sensory impairments after stroke: Self-reported experiences of daily life and rehabilitation. J Rehabil Med. (2018) 50:45–51. 10.2340/16501977-2282 29068038

[4] Bertolucci F Chisari C Fregni F. The potential dual role of transcallosal inhibition in post-stroke motor recovery. Restor Neurol Neurosci. (2018) 36:83–97. 10.3233/RNN-170778 29439366

[5] Talelli P Greenwood RJ Rothwell JC. Arm function after stroke: neurophysiological correlates and recovery mechanisms assessed by transcranial magnetic stimulation. Clin Neurophysiol. (2006) 117:1641–59. 10.1016/j.clinph.2006.01.016 16595189

[6] Traversa R Cicinelli P Pasqualetti P Filippi M Rossini PM. Follow-up of interhemispheric differences of motor evoked potentials from the 'affected' and 'unaffected' hemispheres in human stroke. Brain Res. (1998) 803:1–8. 10.1016/S0006-8993(98)00505-8 9729235

[7] Reithler J Peters JC Sack AT. Multimodal transcranial magnetic stimulation: using concurrent neuroimaging to reveal the neural network dynamics of noninvasive brain stimulation. Prog Neurobiol. (2011) 94:149–65. 10.1016/j.pneurobio.2011.04.004 21527312

[8] McDonnell MN Stinear CM. TMS measures of motor cortex function after stroke: a meta-analysis. Brain Stimul. (2017) 10:721–34. 10.1016/j.brs.2017.03.008 28385535

[9] Rossi S Hallett M Rossini PM Pascual-Leone A. Safety, ethical considerations, and application guidelines for the use of transcranial magnetic stimulation in clinical practice and research. Clin Neurophysiol. (2009) 120:2008–39. 10.1016/j.clinph.2009.08.016 19833552 PMC3260536

[10] Lefaucheur JP Aleman A Baeken C Benninger DH Brunelin J Di Lazzaro V . Evidence-based guidelines on the therapeutic use of repetitive transcranial magnetic stimulation (rTMS): An update (2014-2018). Clin Neurophysiol. (2020) 131:474–528. 10.1016/j.clinph.2019.11.002 31901449

[11] Vuksanović J Jelić MB Milanović SD Kačar K Konstantinović L Filipović SR. Improvement of language functions in a chronic non-fluent post-stroke aphasic patient following bilateral sequential theta burst magnetic stimulation. Neurocase. (2015) 21:244–50. 10.1080/13554794.2014.890731 24579976

[12] Chou YH Hickey PT Sundman M Song AW Chen NK. Effects of repetitive transcranial magnetic stimulation on motor symptoms in Parkinson disease: a systematic review and meta-analysis. JAMA Neurol. (2015) 72:432–40. 10.1001/jamaneurol.2014.4380 25686212 PMC4425190

[13] Graef P Dadalt M Rodrigués D Stein C Pagnussat AS. Transcranial magnetic stimulation combined with upper-limb training for improving function after stroke: a systematic review and meta-analysis. J Neurol Sci. (2016) 369:149–58. 10.1016/j.jns.2016.08.016 27653882

[14] Zhang L Xing G Fan Y Guo Z Chen H Mu Q. Short- and Long-term Effects of Repetitive Transcranial Magnetic Stimulation on Upper Limb Motor Function after Stroke: a Systematic Review and Meta-Analysis. Clin Rehabil. (2017) 31:1137–53. 10.1177/0269215517692386 28786336

[15] Hutton B Salanti G Caldwell DM Chaimani A Schmid CH Cameron C . The PRISMA extension statement for reporting of systematic reviews incorporating network meta-analyses of health care interventions: checklist and explanations. Ann Intern Med. (2015) 162:777–84. 10.7326/M14-2385 26030634

[16] Hatano S. Experience from a multicentre stroke register: a preliminary report. Bull World Health Organ. (1976) 54:541–53. 1088404 PMC2366492

[17] TtCDCotCM A. Various types of diagnosis of cerebrovascular diseases. Chin J Neurol. (1996) 29:60–1.6236049

[18] Kang MG Yun SJ Lee SY Oh BM Lee HH Lee SU . Effects of Upper-Extremity Rehabilitation Using Smart Glove in Patients With Subacute Stroke: Results of a Prematurely Terminated Multicenter Randomized Controlled Trial. Front Neurol. (2020) 11:580393. 10.3389/fneur.2020.580393 33240205 PMC7680863

[19] Liu L Jin M Zhang L Zhang Q Hu D Jin L . Brain-Computer Interface-Robot Training Enhances Upper Extremity Performance and Changes the Cortical Activation in Stroke Patients: A Functional Near-Infrared Spectroscopy Study. Front Neurosci. (2022) 16:809657. 10.3389/fnins.2022.809657 35464315 PMC9024364

[20] Zhan J Ai Y Zhan L Pan R Wang Y Dong C . Effect of abdominal acupuncture combined with routine rehabilitation training on shoulder-hand syndrome after stroke: a randomized controlled trial. Integr Med Res. (2022) 11:100805. 10.1016/j.imr.2021.100805 34877254 PMC8627967

[21] Fang JX Wang EQ Wang W Liu Y Cheng G. The efficacy and safety of high-dose statins in acute phase of ischemic stroke and transient ischemic attack: a systematic review. Intern Emerg Med. (2017) 12:679–87. 10.1007/s11739-017-1650-8 28303440

[22] Higgins JP Altman DG Gøtzsche PC Jüni P Moher D Oxman AD . The Cochrane Collaboration's tool for assessing risk of bias in randomised trials. BMJ. (2011) 343:d5928. 10.1136/bmj.d5928 22008217 PMC3196245

[23] Shim SR Kim SJ Lee J Rücker G. Network meta-analysis: application and practice using R software. Epidemiol Health. (2019) 41:e2019013. 10.4178/epih.e2019013 30999733 PMC6635665

[24] Sheng S Zhao T Wang X. Comparison of robot-assisted surgery, laparoscopic-assisted surgery, and open surgery for the treatment of colorectal cancer: a network meta-analysis. Medicine (Baltimore). (2018) 97:e11817. 10.1097/MD.0000000000011817 30142771 PMC6112974

[25] Wang Q Zhang D Zhao YY Hai H Ma YW. Effects of high-frequency repetitive transcranial magnetic stimulation over the contralesional motor cortex on motor recovery in severe hemiplegic stroke: a randomized clinical trial. Brain Stimul. (2020) 13:979–86. 10.1016/j.brs.2020.03.020 32380449

[26] Yeh KH Tsai TH Chai HT Leu S Chung SY Chua S . Comparison of acute versus convalescent stage high-sensitivity C-Reactive protein level in predicting clinical outcome after acute ischemic stroke and impact of erythropoietin. J Transl Med. (2012) 10:6. 10.1186/1479-5876-10-6 22222005 PMC3286363

[27] Brunelli S Iosa M Fusco FR Pirri C Di Giunta C Foti C . Early body weight-supported overground walking training in patients with stroke in subacute phase compared to conventional physiotherapy: a randomized controlled pilot study. Int J Rehabil Res. (2019) 42:309–15. 10.1097/MRR.0000000000000363 31425349

[28] Avan A Digaleh H Di Napoli M Stranges S Behrouz R Shojaeianbabaei G . Socioeconomic status and stroke incidence, prevalence, mortality, and worldwide burden: an ecological analysis from the Global Burden of Disease Study 2017. BMC Med. (2019) 17:191. 10.1186/s12916-019-1397-3 31647003 PMC6813111

[29] Pollock A Farmer SE Brady MC Langhorne P Mead GE Mehrholz J . Interventions for improving upper limb function after stroke. Cochrane Database Syst Rev. (2014) 2014:CD010820. 10.1002/14651858.CD010820.pub2 25387001 PMC6469541

[30] Kumar A Schmeler MR Karmarkar AM Collins DM Cooper R Cooper RA . Test-retest reliability of the functional mobility assessment (FMA): a pilot study. Disabil Rehabil Assist Technol. (2013) 8:213–9. 10.3109/17483107.2012.688240 22612721

[31] Morris DM Uswatte G Crago JE Cook EW 3rd Taub E. The reliability of the wolf motor function test for assessing upper extremity function after stroke. Arch Phys Med Rehabil. (2001) 82:750–5. 10.1053/apmr.2001.23183 11387578

[32] Hsu WY Cheng CH Liao KK Lee IH Lin YY. Effects of repetitive transcranial magnetic stimulation on motor functions in patients with stroke: a meta-analysis. Stroke. (2012) 43:1849–57. 10.1161/STROKEAHA.111.649756 22713491

[33] Di Pino G Pellegrino G Assenza G Capone F Ferreri F Formica D . Modulation of brain plasticity in stroke: a novel model for neurorehabilitation. Nat Rev Neurol. (2014) 10:597–608. 10.1038/nrneurol.2014.162 25201238

[34] Cassidy JM Chu H Anderson DC Krach LE Snow L Kimberley TJ . A Comparison of Primed Low-frequency Repetitive Transcranial Magnetic Stimulation Treatments in Chronic Stroke. Brain Stimul. (2015) 8:1074–84. 10.1016/j.brs.2015.06.007 26198365 PMC4656059

[35] Harris-Love ML Harrington RM. Non-invasive brain stimulation to enhance upper limb motor practice poststroke: a model for selection of cortical site. Front Neurol. (2017) 8:224. 10.3389/fneur.2017.00224 28611727 PMC5447046

[36] Alawieh A Tomlinson S Adkins D Kautz S Feng W. Preclinical and clinical evidence on ipsilateral corticospinal projections: implication for motor recovery. Transl Stroke Res. (2017) 8:529–40. 10.1007/s12975-017-0551-5 28691140 PMC5802360

[37] Takeuchi N Tada T Matsuo Y Ikoma K. Low-frequency repetitive TMS plus anodal transcranial DCS prevents transient decline in bimanual movement induced by contralesional inhibitory rTMS after stroke. Neurorehabil Neural Repair. (2012) 26:988–98. 10.1177/1545968311433295 22412170

[38] Gao F Wang S Guo Y Wang J Lou M Wu J . Protective effects of repetitive transcranial magnetic stimulation in a rat model of transient cerebral ischaemia: a microPET study. Eur J Nucl Med Mol Imaging. (2010) 37:954–61. 10.1007/s00259-009-1342-3 20107794

[39] Di Lazzaro V Dileone M Pilato F Capone F Musumeci G Ranieri F . Modulation of motor cortex neuronal networks by rTMS: comparison of local and remote effects of six different protocols of stimulation. J Neurophysiol. (2011) 105:2150–6. 10.1152/jn.00781.2010 21346213

[40] Bai Z Zhang J Fong K. Effects of transcranial magnetic stimulation in modulating cortical excitability in patients with stroke: a systematic review and meta-analysis. J Neuroeng Rehabil. (2022) 19:24. 10.1186/s12984-022-00999-4 35193624 PMC8862292

[41] Suppa A Huang YZ Funke K Ridding MC Cheeran B Di Lazzaro V . Ten years of theta burst stimulation in humans: established knowledge, unknowns and prospects. Brain Stimul. (2016) 9:323–35. 10.1016/j.brs.2016.01.006 26947241

[42] Oberman L Edwards D Eldaief M Pascual-Leone A. Safety of theta burst transcranial magnetic stimulation: a systematic review of the literature. J Clin Neurophysiol. (2011) 28:67–74. 10.1097/WNP.0b013e318205135f 21221011 PMC3260517

[43] Chou TY Wang JC Lin MY Tsai PY. Low-frequency vs. theta burst transcranial magnetic stimulation for the treatment of chronic non-fluent aphasia in stroke: a proof-of-concept study. Front Aging Neurosci. (2021) 13:800377. 10.3389/fnagi.2021.800377 35095477 PMC8795082

[44] Chen XW Shafei MN Abdullah JM Musa KI. Reliability of telephone interview for assessment of long-term stroke outcomes: evidence from interrater analysis. Neuroepidemiology. (2019) 52:214–9. 10.1159/000497238 30799411

[45] Branco JP Oliveira S Páscoa Pinheiro J L Ferreira P. Assessing upper limb function: transcultural adaptation and validation of the Portuguese version of the Stroke Upper Limb Capacity Scale. BMC Sports Sci Med Rehabil. (2017) 9:15. 10.1186/s13102-017-0078-9 28785412 PMC5543451

[46] Fujita T Sato A Togashi Y Kasahara R Ohashi T Tsuchiya K . Identification of the affected lower limb and unaffected side motor functions as determinants of activities of daily living performance in stroke patients using partial correlation analysis. J Phys Ther Sci. (2015) 27:2217–20. 10.1589/jpts.27.2217 26311957 PMC4540852

[47] Meyer BC Hemmen TM Jackson CM Lyden PD. Modified National Institutes of Health Stroke Scale for use in stroke clinical trials: prospective reliability and validity. Stroke. (2002) 33:1261–6. 10.1161/01.STR.0000015625.87603.A7 11988601

