# Supplementary material for: Comparative Efficacy of Different Repetitive Transcranial Magnetic Stimulation Protocols for Stroke: A Network Meta-Analysis
Source: Front Neurol. 2022 Jun 15;13:918786. doi: 10.3389/fneur.2022.918786 (PMC9240662; doi:10.3389/fneur.2022.918786)
Supplement: Supplementary file 1 [file Table_1.DOCX]

**Supplementary Material**

**Table of Contents**

**Table1.** The characteristics of included studies.

**Table2.**Quality evaluation of included studies.

**Table3.**The PSRF value.

**Table4.** Node-splitting model assessing incoherence between direct and indirect comparisons.

**Table5.**Summary of adverse reactions.

**Figure1.**Sensitivity analysis of UE-FMA.A:Forest plot of sensitivity analysis.

B:The ranking probability of sensitivity analysis.

**Figure2.** Sensitivity analysis of MBI.A:Forest plot of sensitivity analysis.

B:The ranking probability of sensitivity analysis.

**Figure3.** Sensitivity analysis of NIHSS.A:Forest plot of sensitivity analysis.

B:The ranking probability of sensitivity analysis.

**Figure4.** Subgroup analysis of mild stroke group (UE-FMA score≥33) .A:Forest plot of subgroup analysis.B:The ranking probability of subgroup analysis.

**Figure5**.Subgroup analysis of severe stroke group (UE-FMA score <33) .A:Forest plot of subgroup analysis.B:The ranking probability of subgroup analysis.

**Figure6.** Subgroup analysis of acute phase and subacute phase group (<1 month).A: Forest plot of subgroup analysis.B:The ranking probability of subgroup analysis.

**Figure7.** Subgroup analysis of convalescent phase group (>1 month). A: Forest plot of subgroup analysis.B:The ranking probability of subgroup analysis.

**Table1.** **The characteristics of included studies.**

| Study | Country | Sample size  (E/C) | Age (years) (E/C) | Interventions  (E/C) | No. of pulses | rTMS frequency (Hz) | Onset time  (months) | rTMS site | Outcome measures |
| --- | --- | --- | --- | --- | --- | --- | --- | --- | --- |
| Ackerley 2016^1^ | New Zealand | 9/9 | 21-80/  38-79 | CR+ITBS/  CR+Sham | 600p×10 | ITBS | >6 | Ipsilateral |  |
| Chen  2019^2^ | China | 11/11 | 52.9±11.1/  52.6 ± 8.3 | CR+ITBS/  CR+ Sham | 600p×10 | ITBS | >6 | Ipsilateral |  |
| Lazzaro-  2016^3^ | Italy | 8/9 | 57.9±4.4  /56.7± 3.2 | CR+CTBS/  CR_+_ Sham | 600p×10 | CTBS | >12 | contralateral |  |
| Hsu-  2013^4^ | China | 6/6 | 56.8±6.8/  62.3±8.5 | CR+ITBS/  CR+ Sham | 1200p×10 | ITBS | <1 | Ipsilateral | ①③ |
| Khan-  2019^5^ | India | 20/20 | 63.5±12.6/  64.6±12.9 | ITBS+CR/  CR+ Sham | 600 p×12 | ITBS | <1 | Ipsilateral | ①③④ |
| Koch-  2019^6^ | Italy | 17/17 | 64.0±11.3/  64.9±11.9 | CR+ITBS/  CR+ Sham | 1200 p×15 | ITBS | >6 | Cerebellum | ④ |
| Kondo-  2017^7^ | Japan | 71/32 | 62.3±12.5/  60.0±14.2 | LFrTMS+CR/CR +CTBS | 2400p×10/  2400p×10 | 1Hz/  CTBS | >12 | contralateral |  |
| Nicolo-  2018^8^ | Switzerland | 14/13 | 62.4±12.3/  64.3 ±17.1 | CR+CTBS/  CR+ Sham | NR | CTBS | >1 | contralateral |  |
| Watanabe-  2018^9^ | Japan | 8/7/6 | 72.5±6.5/  67.6±6.4/  75.2±5.5 | CR+ITBS/  CR+LFrTMS/CR+ Sham | 600p×10  /1200p×10 | ITBS/  1Hz | <1 | Ipsilateral/  contralateral |  |
| TangWX-  2018^10^ | China | 8/8 | 53.7±10.7/  55.6±14.5 | CR+ITBS/  CR+ Sham | 600p×10 | ITBS | 1～6 | Ipsilateral | 1. ④ |
| JiangC- 2018^11^ | China | 13/11/13 | 61.3±11.2/  59.2±9.8/  51.8±11.5 | CR+ITBS/  CR+LFrTMS/CR+ Sham | 600p×10  /600p×10 | ITBS/  1Hz | >1 | Ipsilateral/  contralateral | ①④ |
| XiangWP  2017^12^ | China | 18/18/18/18 | 60.4±8.7 60.2±6.8/  59.1±7.2/ 58.9±6.1 | CR+ITBS/  CR+HFrTMS/  CR+LFrTMS  /CR+ Sham | 2000p×14  /2000p×14  /2000p×10 | ITBS/  10Hz/  1Hz | <1 | Ipsilateral/  contralateral |  |
| Sung-  2013^13^ | China | 13/14/15 | 64.2±11.9/  63.3±12.8/  63.1±12.8 | CR+ITBS/  CR+LFrTMS /  CR+ Sham | 600p×10  /600p×10 | ITBS/  1Hz | 3～12 | Ipsilateral/  contralateral | 1. ③ |
| Miao YJ 2020^14^ | China | 22/21 | 55.8±9.1/  59.5±13.1 | CR+ITBS/  CR+ Sham | 600p×12 | ITBS | <1 | Ipsilateral | 1. ④ |
| guo 2016^15^ | China | 7/8 | 67.7±7.4/  66.6±9.2 | HFrTMS+CR/  CR+ Sham | 1500p×10 | 10Hz | <1 | Ipsilateral | 1. ④ |
| Hosomi 2016^16^ | Japan | 20/21 | 62.4±15.5/  63.2±12.5 | HFrTMS+CR/  CR+ Sham | 1500p×10 | 5Hz | <2 | Ipsilateral | ③ |
| Ibrahim 2020^17^ | Egypt | 20/20 | 58.7±5.5/  60.2±5.3 | HFrTMS+CR/  CR+ Sham | 400p×10 | 5Hz | >3 | Ipsilateral | ① |
| Ke 2020^18^ | China | 16/16 | 57.5±7.9/  58.3±8.3 | HFrTMS+CR /  CR+ Sham | 1200p×10 | 20Hz | <1 | Ipsilateral | ①④ |
| Kim 2010^19^ | Korea | 18/10 | 56.4±11.2/  57.0±14.5 | HFrTMS+CR/  CR+ Sham | 1000p×10 | 10Hz | <1 | Ipsilateral | ①④ |
| Wang 2020^20^ | China | 15/15 | 60.5±14.1/  60.4±12.1 | LFrTMS+CR/  CR+ Sham | 1000p×14 | 1Hz | <3 | contralateral | ④ |
| Chen CY 2020^21^ | China | 45/45 | 60.1±4.5/  61.3±4.8 | CR+HFrTMS/  CR+ Sham | 2000p×20 | 12Hz | >1 | Ipsilateral | ④ |
| Chen YJ 2018^22^ | China | 91/89 | 56.8±12.4/  57.5± 13.1 | CR+HFrTMS/  CR | NR×20 | 10Hz | <6 | Ipsilateral | ①④ |
| Chen ZY 2019^23^ | China | 54/46 | 65.4±7.4/  64.5±7.4 | CR+HFrTMS/  CR+ Sham | 1500p×20 | 10Hz | <6 | Ipsilateral | ① |
| Chen ZY 2019^24^ | China | 70/70 | 57.1±5.0/ 56.8±4.8 | CR+HFrTMS /  CR+ Sham | 1500p×20 | 10Hz | >1 | Ipsilateral | ④ |
| Liu JF 2020^25^ | China | 44/44 | 63.1±5.1/  62.4±5.4 | CR+HFrTMS /  CR | 2000p×24 | 10Hz | <6 | Ipsilateral | ①④ |
| Liu Y 2020^26^ | China | 70/70 | 56.3±4.1/  56.1±3.9 | HFrTMS+CR/  CR+ Sham | 1200p×72 | 10Hz | >1 | Ipsilateral | ① |
| Liu Y 2019^27^ | China | 21/20 | 55.4±6.7/  58.1±8.4 | HFrTMS+CR/  CR+ Sham | 1500p×40 | 10Hz | <6 | Ipsilateral | ①④ |
| Pan RR 2018^28^ | China | 20/20 | 18-80 | HFrTMS+CR/  CR+ Sham | 3000p×30 | 10Hz | <3 | Ipsilateral | ① |
| Su CX 2018^29^ | China | 20/20 | 51.1±7.7/  49.8±9.1/ | CR+HFrTMS/  CR+ Sham | China | 21/20 | <6 | Ipsilateral | ① |
| Sun SJ 2018^30^ | China | 35/35 | 59.3±10.4/  56.5±9.2 | CR+HFrTMS/  CR+ Sham | China | 20/20 | NR | Ipsilateral | ③④ |
| Xiao CL 2019^31^ | China | 16/15/17 | 58.7±9.9/  60.4±11.7/  57.6±10.6 | CR+HFrTMS /CR+HFrTMS  CR+ Sham | China | 20/20 | <6 | Ipsilateral | ①④ |
| Xiao CL 2018^32^ | China | 13/14 | 59.1±9.3/  60.9±10.2 | CR+HFrTMS/  CR+ Sham | 900p×10 | 3Hz | <6 | Ipsilateral | ④ |
| Yi MY 2018^33^ | China | 12/13 | 58.5±11.9/  60.1±10.3 | CR+HFrTMS/  CR+ Sham | 2000p×20 | 10Hz | <6 | Ipsilateral | ④ |
| You Q 2015^34^ | China | 20/20 | 49.8 ± 9.9/  50.6 ± 10.1 | CR+HFrTMS/  CR+ Sham | 1000p×40 | 20Hz | <3 | Ipsilateral | ④ |
| You GQ 2017^35^ | China | 30/30/30 | 63.6±12.5/  65.1±15 | CR+HFrTMS/  CR+ Sham | NR×20 | 10Hz | <1 | Ipsilateral | ①②④ |
| Zhang N 2014^36^ | China | 45/48 | 59.3±3.2/  58.5±3.6 | CR+HFrTMS/  CR+ Sham | 500p×20 | 5Hz | <1 | Ipsilateral | ④ |
| Liang QT 2018^37^ | China | 38/38 | 65.5±10.5/  64.3±11.8 | CR+HFrTMS/  CR+ Sham | 900p×40 | 3Hz | <6 | contralateral | ①④ |
| Zhang XH 2016^38^ | China | 50/50 | 64.2±7.3/  62.6±4.4 | CR+HFrTMS/  CR+ Sham | 500p×20 | 5Hz | <1 | Ipsilateral | ③④ |
| Zheng J 2017^39^ | China | 30/30 | 58.9±13.5/  61.9±11.3 | CR+HFrTMS/  CR+ Sham | 1500p×30 | 20Hz | <6 | Ipsilateral | ④ |
| Zhou Z 2020^40^ | China | 30/28 | 59.7±10.4/  61.7±11.4 | CR+HFrTMS/  CR+ Sham | 1200p×15 | 5Hz | <12 | Ipsilateral | ①②④ |
| Mehmet 2016^41^ | Turkey | 10/11 | 55.7±14.9/  64.5±9.3 | LFrTMS+CR /  CR+ Sham | 1500p×10 | 1Hz | >1 | contralateral | ① |
| Wang HB 2017^42^ | China | 22/20 | 61.4± 8.1/  60.2± 6.6 | CR+LFrTMS/  CR+ Sham | 1200p×24 | 1Hz | 1～3 | contralateral | ①③ |
| Abo-2013  ^43^ | Japan | 44/22 | 57.7 ± 12.7/  60.3 ± 10.6 | LFrTMS+CR/  CR+ Sham | 1200p×22 | 1Hz | >12 | contralateral | ①③ |
| Aşkın, -2017^44^ | Turkey | 20/20 | 56.7±11.4  /58.8±12.1 | LFrTMS+ CR / CR+ Sham | 1200p×10 | 1Hz | >6 | contralateral | ① |
| Chervyakov-2015^45^ | Russia | 18/22/12 | 57.03±10.07 | LFrTMS/  HFrTMS/ CR | NR/  NR | 1Hz/  10Hz | 1～36 | contralateral/Ipsilateral | ① |
| Chervyakov-2018^46^ | Russia | 11/13/10 | 54.2 ± 11.1/  58.6 ± 10.4/  61.4 ± 11.4 | LFrTMS+CR /HFrTMS+CR /CR+ Sham | 1200p×10  /200p×10 | 1Hz/  10Hz | 1～12 | contralateral/Ipsilateral | ①④ |
| Du-2018^47^ | China | 18/19/16 | 54 ± 12/  56 ± 9/  56 ± 11 | CR+HFrTMS/  CR+LFrTMS/  CR+ Sham | 1200p×5/  1200p×5 | 10Hz/  1Hz | <1 | contralateral/Ipsilateral | ① |
| Forogh-2017^48^ | Iran | 13/13 | 53-79 | LFrTMS/ CR | 1200p×5 | 1Hz | >1 | contralateral | ① |
| Galvao-2014^49^ | Brazil | 10/10 | 57.4±12.0/  64.6±6.8 | LFrTMS+CR /  CR+ Sham | 1500p×10 | 1Hz | >6 | contralateral | ① |
| Harvey-2018^50^ | America | 132/67 | 59.2±13.3/ 57.6±12.7 | LFrTMS+CR /  CR+ Sham | NR×18 | 1Hz | >3 | contralateral | ① |
| Li-Jing 2016^51^ | China | 42/43/42 | 57.8±12.8/  54.0±13.4/ 53.1±13.7 | CR+LFrTMS/  CR+HFrTMS/  CR+ Sham | 1000p×10  /1350p×10 | 1Hz/10Hz | >1 | contralateral/Ipsilateral | ①② |
| Khedr-2009  ^52^ | Egypt | 13/13/13 | 54.7 ± 9.7/  59.0 ± 13.5/  60.0 ± 9.5 | CR+LFrTMS/  CR+HFrTMS/  CR+ Sham | 900p×NR  /900 p×NR | 1Hz/  3Hz | <1 | contralateral/Ipsilateral | ③ |
| Kim-2010  ^53^ | Korea | 6/6/6/6 | 68.3 ± 7.4/  53.5 ± 16.9/  66.8 ± 17.2 | LFrTMS/  HFrTMS /  CR | 900p×10  /450p×10 | 1Hz/  10Hz | >1 | contralateral/Ipsilateral | ④ |
| Li-2016^54^ | China | 7/5 | 30-76 | HFrTMS+CR /  CR+ Sham | NR×10 | 5Hz | <1 | Ipsilateral | ①③④ |
| Long-2018  ^55^ | China | 21/20 | 57±11.78/  56.8±5.4 | LFrTMS+CR/  CR+ Sham | 1000p×15 | 1Hz | <1 | contralateral/Ipsilateral | ①② |
| Lüdemann-Podubecká-2015^56^ | Germany | 20/20 | 65.7±9.9/  68.3±10.8 | LFrTMS+CR/  CR+ Sham | 900p×15 | 1Hz | <6 | contralateral | ② |
| Matsuura-2015^57^ | Japan | 10/10 | 72.2 ± 6.0 /  74.7 ± 12.7 | LFrTMS/ CR | 1200p×5 | 1Hz | <1 | contralateral | ① |
| Meng-2017  ^58^ | China | 10/10 | 64.8±9.5/  65.2±9.7 | LFrTMS+CR /CR+ Sham | 1800p×14 | 1Hz | NR | contralateral | ①③④ |
| Niimi-2020  ^59^ | Japan | 62/33 | 62.3±11.0/  66.2±10.8 | CR+LFrTMS/  CR+ Sham | 1200p×22 | 1Hz | >1 | contralateral | ① |
| Rose-2014  ^60^ | America | 9/10 | 64.7±7.0/  64.6±9.0 | CR+LFrTMS /  CR+ Sham | 1200p×16 | 1Hz | >6 | contralateral | ① |
| Zhao-  2017^61^ | China | 8/9 | 52.7±12.3  /50.5±13.1 | LFrTMS+CR/  CR+ Sham | 1200p×14 | 1Hz | <6 | contralateral | ①④ |
| Seniów-2012^62^ | Poland | 20/20 | 63.5±8.9/  63.4±9.2 | CR+LFrTMS /  CR+ Sham | 1800p×15 | 1Hz | <3 | contralateral | ①②③ |
| Sharma-2020^63^ | India | 47/49 | 54.8±13.4 /52.8±14.9 | CR+LFrTMS /  CR+ Sham | 750p×10 | 1Hz | <1 | contralateral | ①③④ |
| Tosun-2017  ^64^ | Turkey | 9/9 | 57.6±12.6/  61.3±10.1 | LFrTMS+CR /  CR+ Sham | 1200p×20 | 1Hz | <2 | contralateral | ①④ |
| Vaziri-2014  ^65^ | Iran | 6/6 | 55.2±5.4/  57.0±8.6 | LFrTMS+CR/  CR+ Sham | NR×10 | 1Hz | >8 | contralateral | ①④ |
| Kim-2020  ^66^ | Korea | 36/37 | 61.2±11.2/  62.9±13.1 | LFrTMS+CR/  CR+ Sham | 1800p×10 | 1Hz | <1 | contralateral | ①④ |
| Yang-2016  ^67^ | China | 20/20 | 60.7±12.2/  58.7±12.7 | LFrTMS+CR /  CR+ Sham | 900p×10 | 1Hz | >6 | contralateral | ①④ |
| zheng-2015  ^68^ | China | 55/53 | 65.4±13.5 /  66.2±13.1 | LFrTMS+CR/  CR+ Sham | 1800p×24 | 1Hz | <1 | contralateral | ①②④ |
| Guan-2017  ^69^ | China | 21/21 | 59.7±6.8/  57.4±14.0 | HFrTMS+CR/  CR+ Sham | 1000p×10 | 5Hz | <1 | Ipsilateral | ①③④ |
| Cha-2017  ^70^ | Korea | 12/13 | 63.9±8.5/  62.0±8.3 | LFrTMS+CR /  CR+ Sham | 1200p×20 | 1Hz | <6 | contralateral | ① |
| Du-  2016^71^ | China | 23/23/23 | 56.7±8.4/  56.7±12.4/  53.6±13.5 | HFrTMS+CR /LFrTMS+CR/CR+ Sham | 1200p×5 /1200p×5 | 3Hz/  1Hz | <1 | Ipsilateral/contralateral | ①③④ |
| Kim-  2020^72^ | Korea | 8/12 | 67.0±12.9/ 62.1±16.2 | LFrTMS+CR /  CR+ Sham | 1200p×15 | 1Hz | <1 | contralateral | ①④ |
| Zhao LN 2015^73^ | China | 20/20 | 57.7±9.5/  55.5±13.2 | CR+LFrTMS /  CR+ Sham | 1200p×15 | 1Hz | 1～3 | contralateral | ① |
| Fu K 2013^74^ | China | 25/25 | 52.4±10.6/  53.4±8.0 | CR+LFrTMS /  CR+ Sham | NR×20 | 1Hz | NR | contralateral | ①④ |
| Ge YC 2009^75^ | China | 10/10 | 61.3±7.6 | CR+LFrTMS/  CR+ Sham | 1200p×10 | 1Hz | <1 | contralateral | ③④ |
| Wang H 2018^76^ | China | 56/56 | 56.8±7.1/  57.1±7.1 | CR+ LFrTMS  / CR+ Sham | 600p×20 | 1Hz | NR | contralateral | ④ |
| Liao GH  2017^77^ | China | 35/35 | 67.3±5.2/  66.3±5.2 | CR+LFrTMS  / CR+ Sham | 600p×10 | 1Hz | <1 | Ipsilateral | ④ |
| Liu Y 2018^78^ | China | 10/13 | 56.9±9.1/  55.4±8.4 | CR+LFrTMS/  CR+ Sham | 1200p×40 | 1Hz | >3 | contralateral | ①④ |
| Liu SH 2019^79^ | China | 20/20 | 61.3±9.4/  55.0±11.8 | CR +LFrTMS  / CR+ Sham | 1200p×24 | 1Hz | 0.5～6 | contralateral | ①④ |
| Zhang JJ 2019^80^ | China | 15/15 | 52.8±13.0/  53.2±11.8 | CR+ LFrTMS  / CR+ Sham | 1000p×10 | 1Hz | <1 | contralateral | ① |
| Yang YF 2019^81^ | China | 30/30 | 38.7±19.8 | CR+LFrTMS/  CR+ Sham | NR×14 | 1Hz | NR | Ipsilateral | ③ |
| Ren ZS 2018^82^ | China | 43/43 | 58.6±2.1/  58.9±2.2 | CR+ LFrTMS  / CR+ Sham | 1200p×24 | 1Hz | >2 | contralateral | ①② |
| Lu C 2018^83^ | China | 36/36 | 60.9±15.2/  67.9 ±16.9 | CR+LFrTMS/  CR+ Sham | 420p×24 | 1Hz | <6 | contralateral | ①④ |
| Zhang Y 2019^84^ | China | 28/28 | 53.8±8.5/  54.8±7.4 | CR+LFrTMS/  CR+ Sham | 1200p×20 | 1Hz | <3 | contralateral | ①④ |
| Sun W 2017^85^ | China | 19/19 | 55.1±8.5/  53.5±7.9 | CR+LFrTMS/  CR+ Sham | 1200p×24 | 1Hz | >1 | contralateral | ①④ |
| Wu L 2019^86^ | China | 15/15 | 53.2±1.2/  53.7±1.2 | CR+LFrTMS/  CR+ Sham | NR×20 | 1Hz | NR | contralateral | ④ |
| Cui HC 2017^87^ | China | 21/21 | 53.3±9.7/  54.4±9.1 | CR+LFrTMS/  CR+ Sham | 800p×20 | 1Hz | >1 | contralateral | ①④ |
| Li Q 2018^88^ | China | 10/10 | 63.2±8.9/64.9±8 | CR+LFrTMS/  CR+ Sham | 224p×20 | 1Hz | >1 | contralateral | ① |
| Lin MT 2017^89^ | China | 30/30 | 62.7±4.1/  61.8±2.9 | CR+LFrTMS/  CR+ Sham | NR×10 | 1Hz | <1 | contralateral | ④ |
| Wang HL 2010^90^ | China | 30/30 | 56.2±5.4/  57.2±6.8 | CR+LFrTMS/  CR+ Sham | 500p×14 | 1Hz | >6 | contralateral | ④ |
| Xiang WP 2015^91^ | China | 28/27 | 52.2/53.8 | CR+LFrTMS/  CR+ Sham | 2000p×10 | 1Hz | >6 | Ipsilateral | ① |
| Zhao L 2017^92^ | China | 15/15 | 55.8±13.6/  51.1±8.5 | CR+LFrTMS/  CR+ Sham | 1200p×18 | 1Hz | >12 | contralateral | ④ |
| Zhao XL 2018^93^ | China | 36/39 | 54±11.4/  56±12.7 | CR+LFrTMS/  CR+ Sham | 1000p×20 | 1Hz | <1 | contralateral | ① |
| Zhou WL 2020^94^ | China | 50/50 | 58.8±7.5/  58.3±7.6 | CR+LFrTMS /  CR+ Sham | NR×15 | 1Hz | <1 | Ipsilateral | ① |
| Xu H 2013^95^ | China | 15/15/15 | 58.2±8.1/  58.4±8.6/  62.8±6.3 | CR+LFrTMS /CR+HFrTMS / CR+ Sham | 2000p×14  /2000p×14 | 1Hz/  10Hz | >6 | contralateral/Ipsilateral | ③④ |

①UE-FMA=Upper Extremity Fugl-Meyer Assessment;②WMFT=Wolf Motor Function Test;③NIHSS=National Institutes of Health Stroke scale;④MBI=modified Barthel index;rTMS=repetitive transcranial magnetic stimulation;CR=conventional rehabilitation;Sham=sham rTMS;ITBS:intermittent theta-burst stimulation;CTBS=

continuous theta-burst stimulation;LF-rTMS=lowfrequency repetitive transcranial magnetic stimulation;HF-rTMS= :highfrequency repetitive transcranial magnetic stimulation；NR=Not reported;NO=No adverse reactions;YES=Have adverse reactions;E=experimental group,C=control group.

**Table2.Quality evaluation of included studies.**

| Author | Sequence generation | Allocation  Concealment | Blinding participants and personnel | Blinding ofoutcome data | Incomplete outcome data | Selective outcome reporting | Overall risk of bias |
| --- | --- | --- | --- | --- | --- | --- | --- |
| Abo-2013 | Unclear | Unclear | Unclear | Low | Low | Unclear | Unclear |
| Ackerley 2015 | Low | Low | Low | Low | Low | Unclear | Unclear |
| Aşkın, 2017 | Low | Low | Unclear | Low | Low | Unclear | Unclear |
| Cha2017 | Unclear | Unclear | Low | Low | Low | Unclear | Unclear |
| Chen2019 | Unclear | Unclear | Low | Low | Low | Low | Unclear |
| Chen CY2020 | Low | Unclear | Unclear | Unclear | Low | Unclear | Unclear |
| Chen YJ 2018 | Low | Unclear | Unclear | Unclear | Low | Unclear | Unclear |
| Chen ZY 2019 | Low | Unclear | Unclear | Unclear | Low | Unclear | Unclear |
| Chen ZY 2019A | Low | Unclear | Unclear | Unclear | Low | Unclear | Unclear |
| Chervyakov-2015 | Unclear | Unclear | Low | Unclear | High | Unclear | Unclear |
| Chervyakov-2018 | Low | Low | Unclear | Low | Low | Low | Unclear |
| Cui HC2017 | Unclear | Low | Unclear | Unclear | Low | Unclear | Unclear |
| Du-2016 | Low | Unclear | Unclear | Unclear | Low | Unclear | Unclear |
| Du-2018 | Low | Low | Low | Low | Low | Low | Unclear |
| Forogh-2017 | Unclear | Unclear | Low | Unclear | Low | Unclear | Unclear |
| Fu K 2013 | Low | Low | Low | Low | Low | Unclear | Unclear |
| Galvao-2014 | Low | Low | Low | Low | Low | Unclear | Unclear |
| Ge YC 2009 | Low | Low | Low | Low | Low | Unclear | Unclear |
| Guan-2017 | Low | Low | Low | Low | Low | Low | Unclear |
| guo 2016 | Unclear | Unclear | Low | Low | Low | Unclear | Unclear |
| Harvey-2018 | Low | Low | Low | Low | Low | Unclear | Unclear |
| Hosomi 2016 | Low | Unclear | Low | Low | Low | Low | Unclear |
| Hsu-2013 | Unclear | Unclear | Low | Low | Low | Low | Unclear |
| Ibrahim 2020 | Unclear | Unclear | Low | Low | Low | Unclear | Unclear |
| Jiang C 2018 | Low | Unclear | Low | Low | Low | Unclear | Unclear |
| Ke 2020 | Low | Unclear | Low | Low | Low | Unclear | Unclear |
| Khan-2019 | Low | Low | Low | Low | Low | Low | Unclear |
| Khedr-2009 | Unclear | Unclear | Low | Low | Low | Unclear | Unclear |
| Kim2010 | Low | Unclear | Low | Low | Low | Unclear | Unclear |
| Kim-2010 | Unclear | Unclear | Low | Low | Low | Unclear | Unclear |
| Kim-2020 | Low | Low | Low | Low | Low | Low | Unclear |
| Kim-2020A | Low | Low | Low | Low | Low | Low | Unclear |
| Koch-2018 | Low | Low | Low | Low | Low | Low | Unclear |
| Kondo-2017 | Unclear | Unclear | Unclear | Unclear | Low | Unclear | Unclear |
| Lazzaro2016 | Low | Unclear | Low | Low | Low | Unclear | Unclear |
| Li2016 | Unclear | Unclear | Unclear | Unclear | Low | Unclear | Unclear |
| Liang QT 2018 | Low | Unclear | Unclear | Low | Low | Unclear | Unclear |
| Liao GH2017 | Low | Unclear | Unclear | Low | Low | Unclear | Unclear |
| Liao YJ 2020 | Unclear | Unclear | Unclear | Unclear | Low | Unclear | Unclear |
| Li-Jing 2016 | Unclear | Unclear | Unclear | Unclear | Low | Unclear | Unclear |
| Lin MT 2017 | Low | Unclear | Unclear | Unclear | Low | Unclear | Unclear |
| Li Q 2018 | Low | Unclear | Unclear | Unclear | Low | Unclear | Unclear |
| Liu JF 2020 | Low | Unclear | Unclear | Unclear | Low | Unclear | Unclear |
| Liu SH 2019 | Unclear | Unclear | Unclear | Unclear | Low | Unclear | Unclear |
| Liu Y 2020 | Low | Unclear | Unclear | Unclear | Low | Unclear | Unclear |
| Liu Y 2018 | Low | Unclear | Unclear | Unclear | Low | Unclear | Unclear |
| Liu Y 2019 | Low | Unclear | Unclear | Unclear | Low | Unclear | Unclear |
| Long-2018 | Unclear | Unclear | Low | Low | Low | Unclear | Unclear |
| Lu C 2018 | Low | Unclear | Unclear | Unclear | Low | Unclear | Unclear |
| Lüdemann-Podubecká-2015 | Low | Low | Low | Unclear | Low | Unclear | Unclear |
| Matsuura-2015 | Unclear | Unclear | Low | Low | Low | Low | Unclear |
| Mehmet 2016 | Low | Low | Low | Low | Low | Unclear | Unclear |
| Meng-2017 | Unclear | Unclear | Unclear | Unclear | Low | Unclear | Unclear |
| Nicolo-2017 | Unclear | Unclear | Unclear | Unclear | Low | Unclear | Unclear |
| Niimi-2020 | Unclear | Unclear | Unclear | Unclear | Low | Unclear | Unclear |
| Pan RR 2018 | Low | Unclear | Unclear | Unclear | Low | Unclear | Unclear |
| Ren ZS 2018 | Low | Low | Low | Low | Low | Unclear | Unclear |
| Rose-2014 | Unclear | Unclear | Low | Low | Low | Unclear | Unclear |
| Seniów-2012 | Low | Low | Low | Unclear | Low | Unclear | Unclear |
| Sharma-2020 | Low | Low | Low | Low | Low | Unclear | Unclear |
| Su CX 2018 | Low | Unclear | Unclear | Unclear | Low | Unclear | Unclear |
| Sung-2013 | Low | Low | Low | Unclear | Low | Unclear | Unclear |
| Sun SJ 2018 | Unclear | Unclear | Unclear | Unclear | Low | Unclear | Unclear |
| Sun W 2017 | Unclear | Unclear | Low | Low | Low | Unclear | Unclear |
| TangWX-2018 | Low | Unclear | Unclear | Unclear | Low | Unclear | Unclear |
| Tosun-2017 | Low | Low | Unclear | Low | Low | Unclear | Unclear |
| Vaziri-2014 | Unclear | Unclear | Unclear | Unclear | Low | Unclear | Unclear |
| Wang 2020 | Unclear | Unclear | Low | Low | Low | Low | Unclear |
| Wang HB 2017 | Low | Low | Unclear | Unclear | Low | Unclear | Unclear |
| Wang H 2018 | Low | Unclear | Unclear | Unclear | Low | Unclear | Unclear |
| Wang HL 2010 | Unclear | Unclear | Unclear | Unclear | Low | Unclear | Unclear |
| Watanabe-2018 | Low | Unclear | Unclear | Low | Low | Low | Unclear |
| Wu L 2019 | Unclear | Unclear | Unclear | Unclear | Low | Unclear | Unclear |
| Xiang WP 2015 | Low | Unclear | Unclear | Unclear | Low | Unclear | Unclear |
| Xiang WP 2017 | Low | Unclear | Unclear | Unclear | Low | Unclear | Unclear |
| Xiao CL 2018 | Low | Unclear | Unclear | Unclear | Low | Unclear | Unclear |
| Xiao CL 2019 | Low | Unclear | Unclear | Unclear | Low | Unclear | Unclear |
| Xu H 2013 | Low | Unclear | Unclear | Unclear | Low | Unclear | Unclear |
| Yang-2016 | Low | Low | Low | Unclear | Low | Low | Unclear |
| Yang YF 2019 | Unclear | Unclear | Unclear | Unclear | Low | Unclear | Unclear |
| Yi MY 2018 | Low | Unclear | Unclear | Unclear | Low | Unclear | Unclear |
| You GQ 2017 | Unclear | Unclear | Unclear | Unclear | Low | Unclear | Unclear |
| You Q 2015 | Unclear | Unclear | Unclear | Unclear | Low | Unclear | Unclear |
| Zhang JJ 2019 | Low | Unclear | Unclear | Low | Low | Unclear | Unclear |
| Zhang N 2014 | Low | Unclear | Unclear | Unclear | Low | Unclear | Unclear |
| Zhang XH 2016 | Low | Unclear | Unclear | Unclear | Low | Unclear | Unclear |
| Zhang Y 2019 | Low |  | Unclear | Unclear | Low | Unclear | Unclear |
| Zhao-2017 | Low | Low | Low | Unclear | Low | Unclear | Unclear |
| Zhao LN 2015 | Low | Low | Low | Unclear | Low | Unclear | Unclear |
| Zhao L 2017 | Low | Unclear | Unclear | Unclear | Low | Unclear | Unclear |
| Zhao XL 2018 | Unclear | Unclear | Unclear | Unclear | Low | Unclear | Unclear |
| Zheng-2015 | Low | Low | Low | Unclear | Low | Unclear | Unclear |
| Zhen J 2017 | Unclear | Unclear | Unclear | Low | Low | Unclear | Unclear |
| Zhou WL 2020 | Unclear | Unclear | Unclear | Unclear | Low | Unclear | Unclear |
| Zhou Z 2020 | Unclear | Unclear | Unclear | Low | Low | Unclear | Unclear |

**Table3.The PSRF value.**

| Parameter | PSRF |
| --- | --- |
| **UE-FMA** | 1.00 |
| d.Placebo.ITBS | 1.00 |
| d.Placebo.CTBS | 1.00 |
| d.Placebo.1Hz rTMS | 1.00 |
| d. Placebo.3-5Hz rTMS | 1.00 |
| d.Placebo.≥10Hz rTMS | 1.00 |
| Sd.d | 1.00 |
| **WMFT** |  |
| d.Placebo.ITBS | 1.01 |
| d. Placebo.1Hz rTMS | 1.01 |
| d. Placebo.3-5Hz rTMS | 1.03 |
| d.Placebo.≥10Hz rTMS | 1.00 |
| d.1HzrTMS.CTBS | 1.00 |
| Sd.d | 1.01 |
| **MBI** |  |
| d.Placebo.ITBS | 1.00 |
| d.Placebo.1Hz rTMS | 1.00 |
| d.Placebo.3-5Hz rTMS | 1.00 |
| d.Placebo.≥10Hz rTMS | 1.00 |
| Sd.d | 1.00 |
| **NIHSS** |  |
| d.Placebo.ITBS | 1.00 |
| d. Placebo.1Hz rTMS | 1.00 |
| d. Placebo.3-5Hz rTMS | 1.00 |
| d.Placebo.≥10Hz rTMS | 1.00 |
| Sd.d | 1.00 |

**Table4. Node-splitting model assessing incoherence between direct and indirect comparisons.**

| Name | Direct effect | Indirect effect | Overall | P-Value |
| --- | --- | --- | --- | --- |
| **UE-FMA** |  |  |  |  |
| CR VS CTBS | 3.28 (-6.51, 13.40) | 6.01 (-5.83, 17.79) | 4.17 (-3.32, 11.88) | 0.74 |
| CR VS 1HzrTMS | 5.03 (3.09, 6.97) | 2.36(-12.73, 17.93) | 4.89 (3.05, 6.79) | 0.72 |
| ITBS VS 1HzrTMS | -4.44 (-14.36, 4.99) | -3.41 (-9.11, 2.14) | -3.32 (-8.49, 1.90) | 0.83 |
| CTBS VS 1HzrTMS | -0.88(-12.85,10.72) | 1.65 (-8.70, 11.67) | 0.74 (-7.08, 8.30) | 0.75 |
| 1HzrTMS VS  3-5HzrTMS | -2.35(-16.02, 11.13) | 1.00 (-4.94, 6.86) | 0.66 (-4.78, 5.91) | 0.65 |
| 1HzrTMS VS  ≥10HzrTMS | 4.11 (-1.41, 9.47) | 4.21 (-0.02, 8.24) | 4.57 (1.32, 7.75) | 0.98 |
| 3-5HzrTMS VS  ≥10Hz rTMS | 0.76 (-12.99, 14.50) | 4.39 (-1.56, 10.45) | 3.88 (-1.91, 9.64) | 0.63 |
| **WMFT** |  |  |  |  |
| 1HzrTMS VS  ≥10HzrTMS | 0.03 (-4.22, 4.56) | 6.46 (-3.35, 15.00) | 0.37 (-2.98, 4.84) | 0.21 |
| **MBI** |  |  |  |  |
| ITBS VS 1HzrTMS | -1.37(-18.61, 16.02) | 0.98 (-5.29, 7.22) | 0.48 (-5.73, 6.54) | 0.8 |
| 1HzrTMS VS  ≥10HzrTMS | 3.15 (-3.66, 9.98) | 2.70 (-1.53, 7.06) | 2.78 (-0.86, 6.51) | 0.9 |
| 3-5HzrTMS VS  ≥10HzrTMS | 0.31 (-15.34, 15.87) | 6.62 (0.50, 12.80) | 5.73 (-0.12, 11.48) | 0.44 |
| **NIHSS** |  |  |  |  |
| ITBS VS 1HzrTMS | 1.63 (-1.60, 4.96) | 2.29 (-0.05, 4.56) | 1.90 (-0.25, 4.02) | 0.72 |
| ITBS VS ≥10HzrTMS | 3.58 (0.44, 6.87) | 1.89 (-0.60, 4.44) | 2.32 (0.13, 4.65) | 0.37 |
| 1HzrTMS VS  ≥10HzrTMS | 0.69 (-1.69, 3.11) | 0.41 (-1.52, 2.51) | 0.42 (-1.10, 2.01) | 0.83 |

**Table5.Summary of adverse reactions.**

| **Study** | Adverse reactions |
| --- | --- |
| Xiang WP-2017 | 8 cases had headache |
| Miao YJ-2020 | 1 case of mild headache, no other ills |
| Chervyakov-2018 | 2 cases of epilepsy |
| Li-Jing 2016 | Muscle numbness |
| Meng-2017 | Only one case of dizziness |
| Sharma-2020 | One had epileptic seizures, which was later ruled out without other ills |
| zheng-2015 | Dizziness, nausea, mild malaise |
| Zhao LN-2015 | One patient developed mild dizziness and vomiting after the first treatment |
| GE YC-2009 | 1 case of headache, 1 case of scalp discomfort |
| Ren ZS-2018 | 1 case of scalp discomfort |
| Zhang Y 2019 | 1 case had a light headache |
| Sun W 2017 | 1 case had a light headache |
| Xue H 2013 | 3 cases had headache and burning skin |


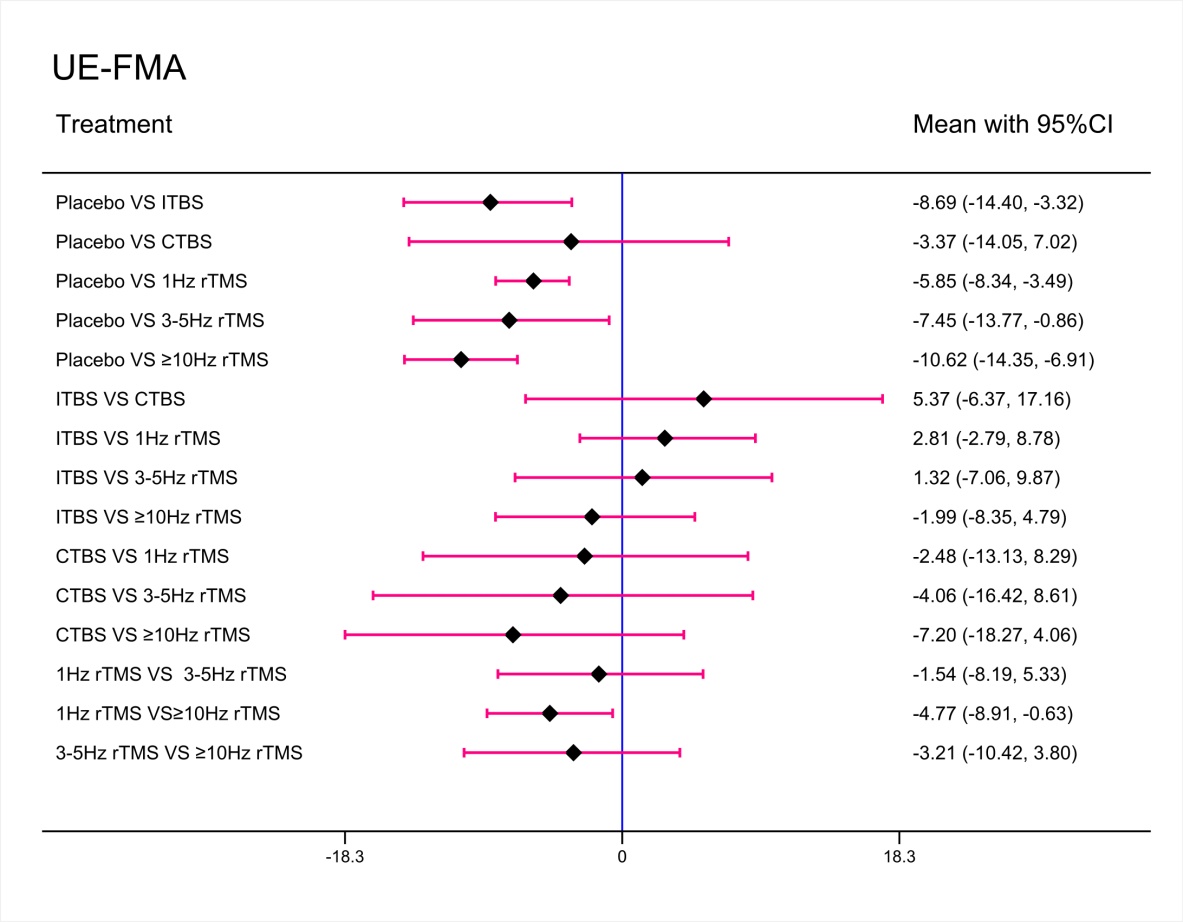
A

**B**

**
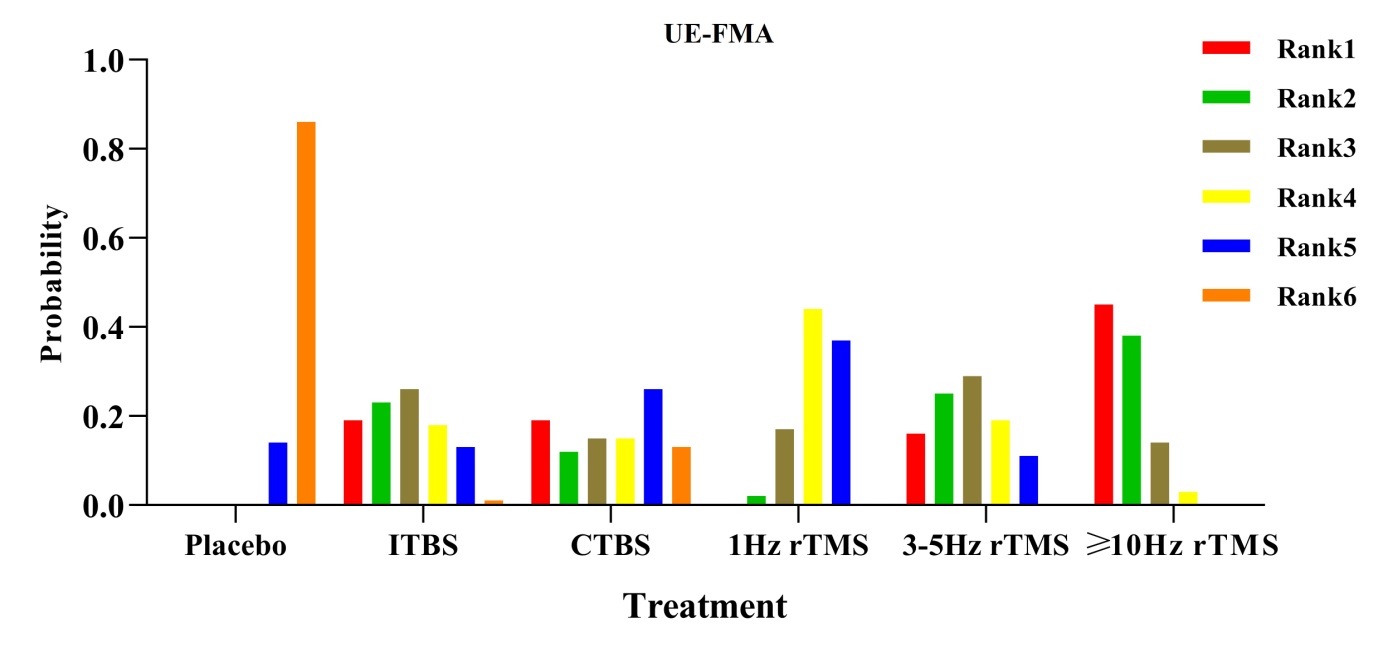
Figure1.** Sensitivity analysis of UE-FMA.A:Forest plot of sensitivity analysis.

B:The ranking probability of sensitivity analysis.

**A**


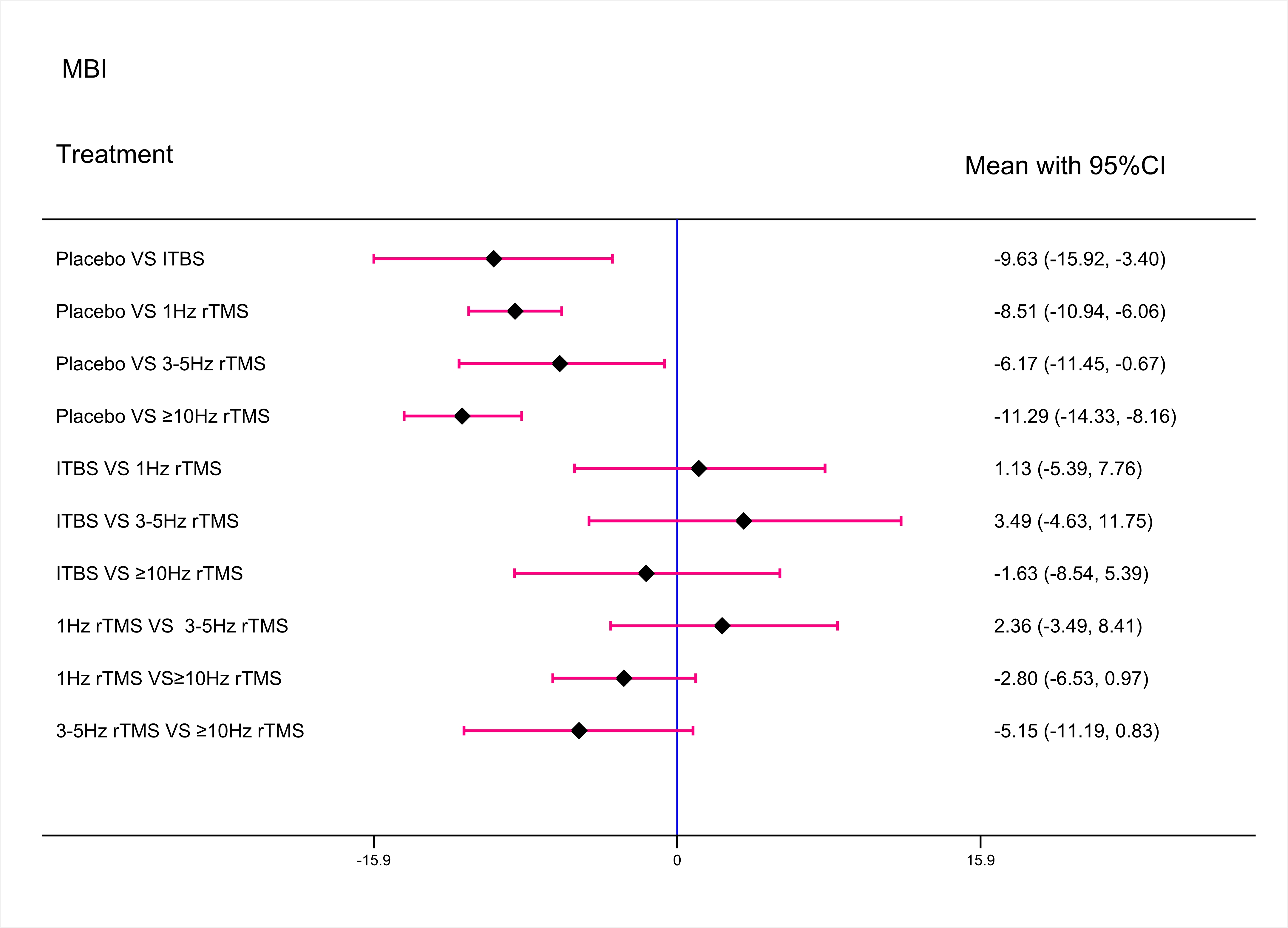


**B**


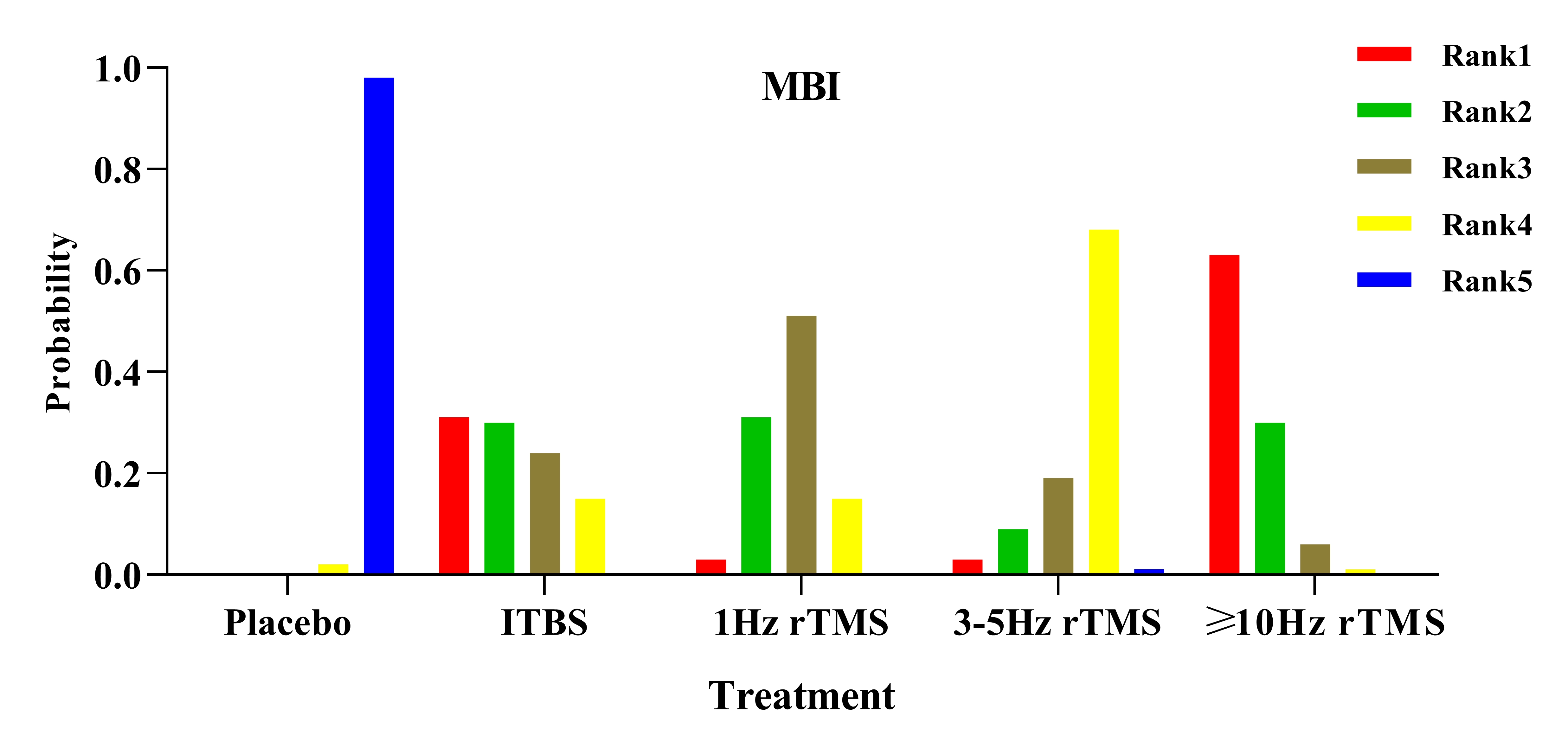


**Figure2.** Sensitivity analysis of MBI.A:Forest plot of sensitivity analysis.

B:The ranking probability of sensitivity analysis.


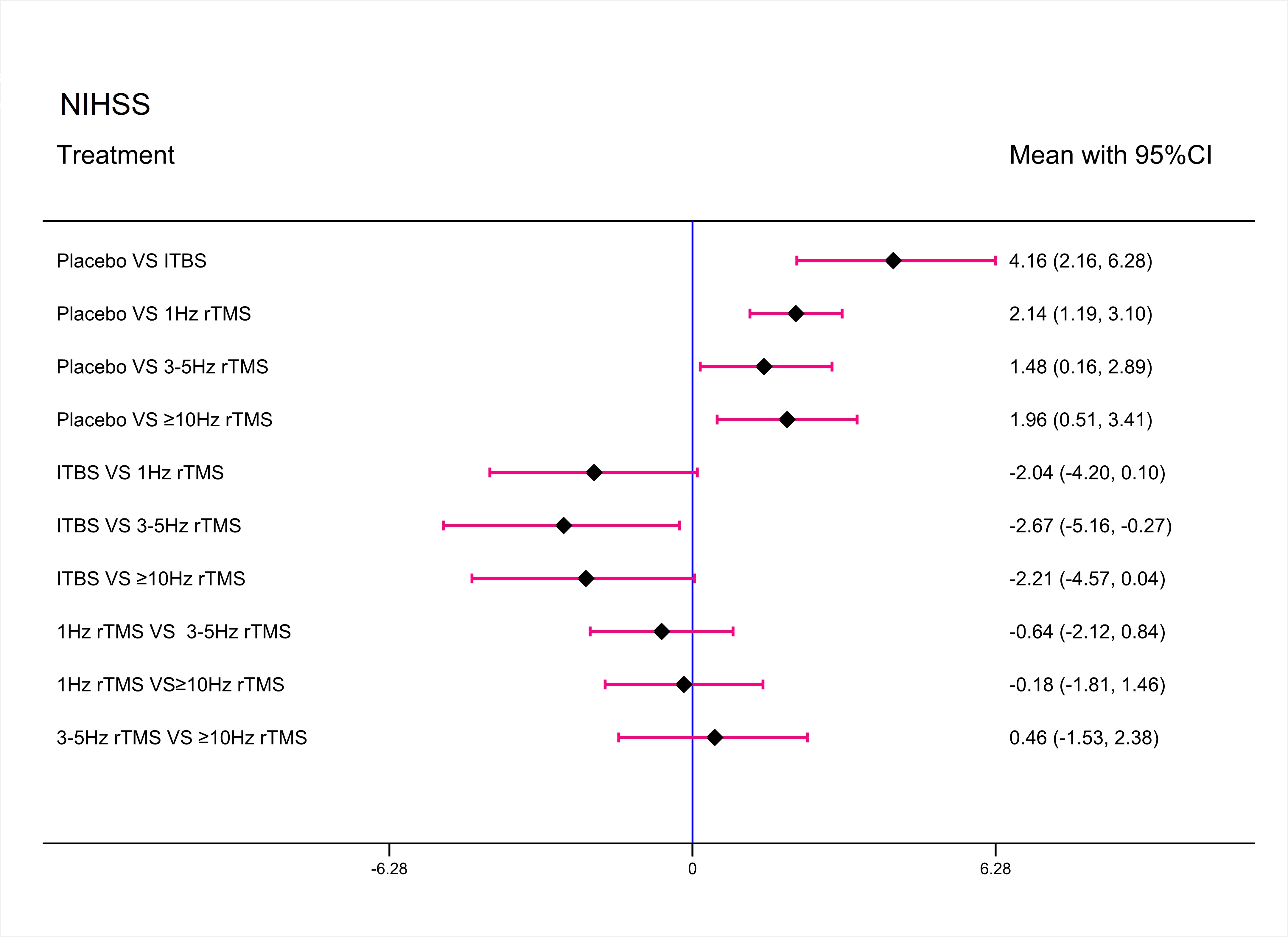


**A**

**B**


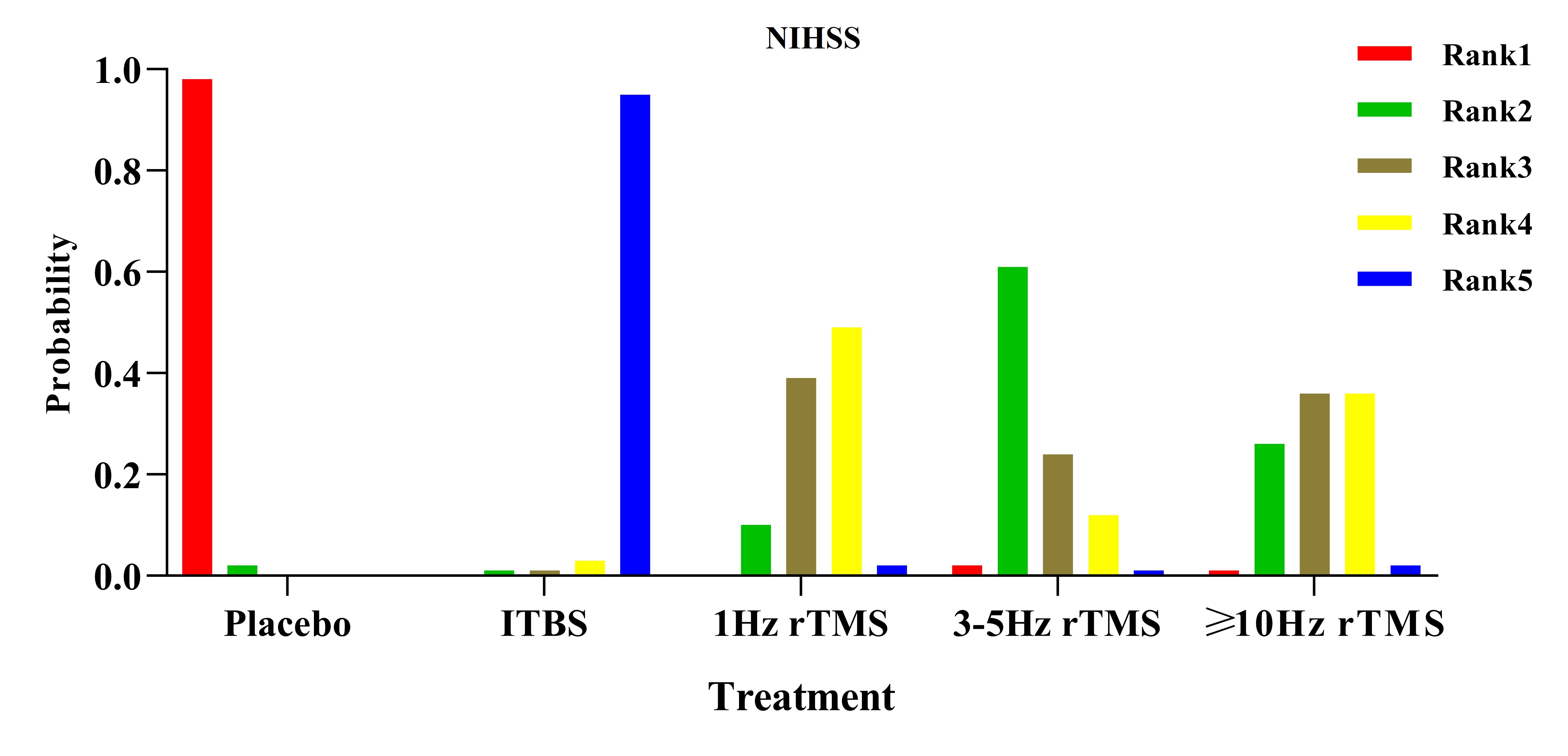


**Figure3.** Sensitivity analysis of NIHSS.A:Forest plot of sensitivity analysis.

B:The ranking probability of sensitivity analysis.


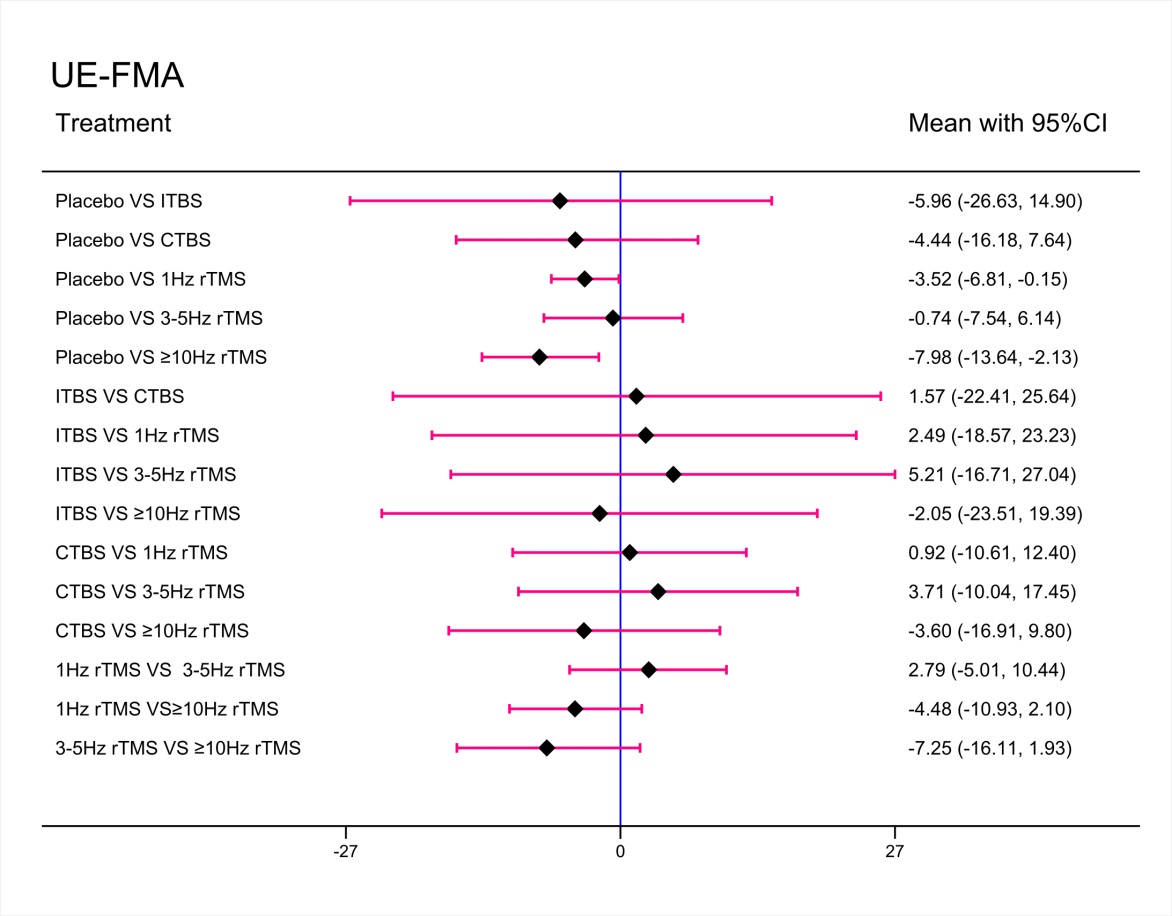
**A**


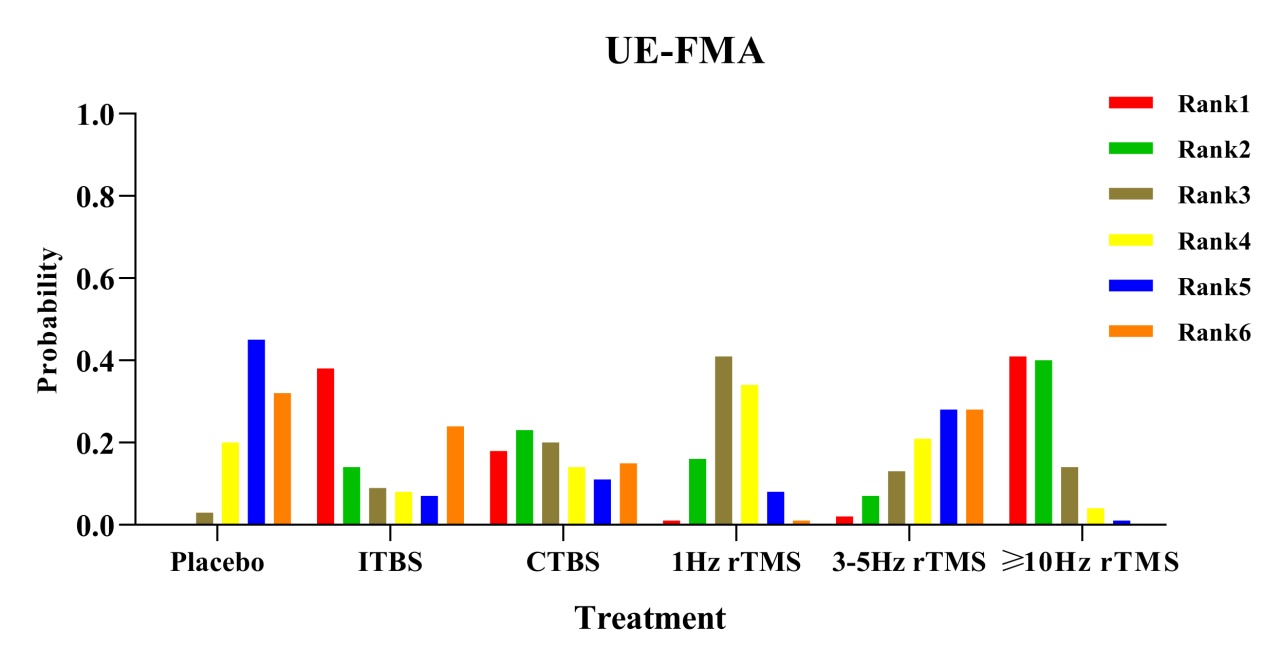
**B**

**Figure4.**Subgroup analysis of mild stroke group (UE-FMA score≥33) .A:Forest plot of subgroupanalysis.B:The ranking probability of subgroup analysis.

**
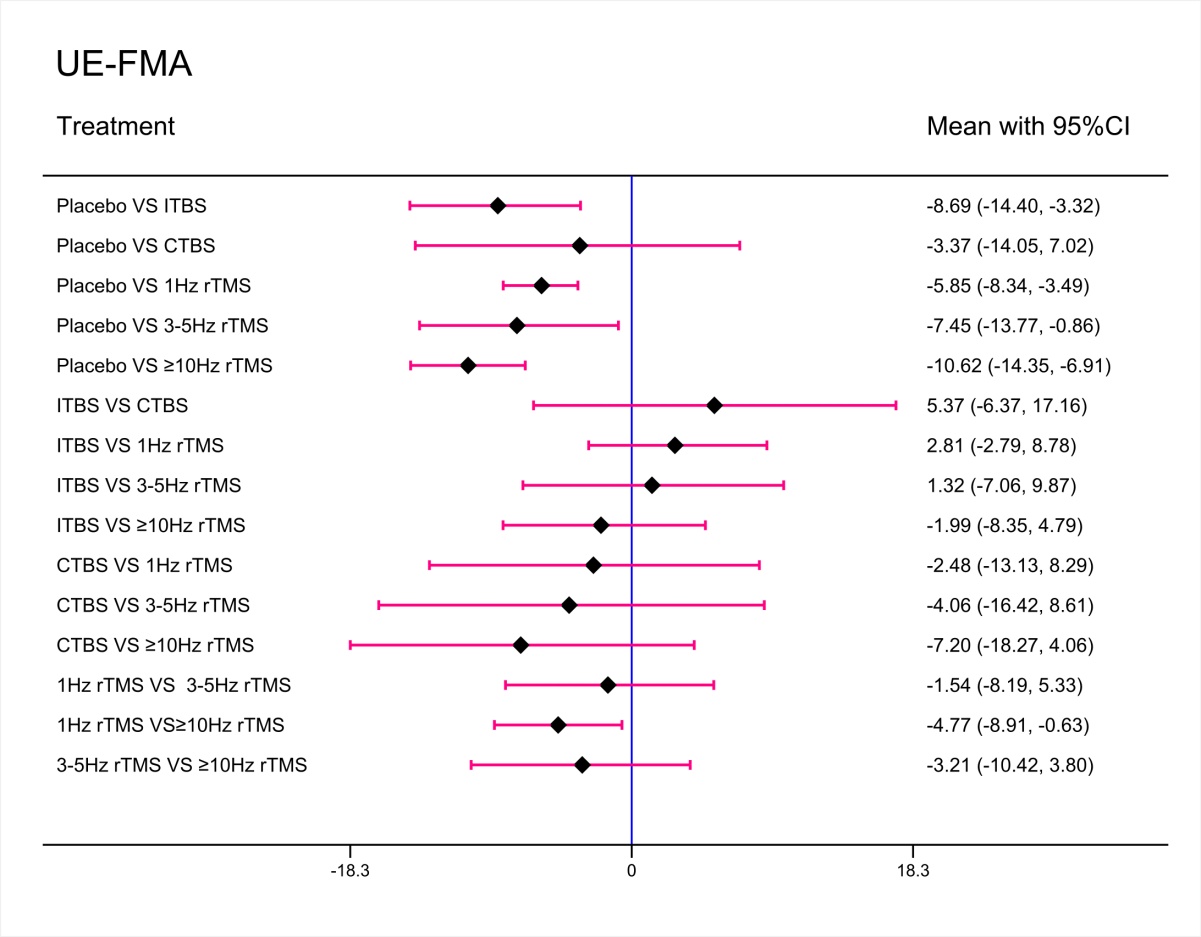
A**

**B**


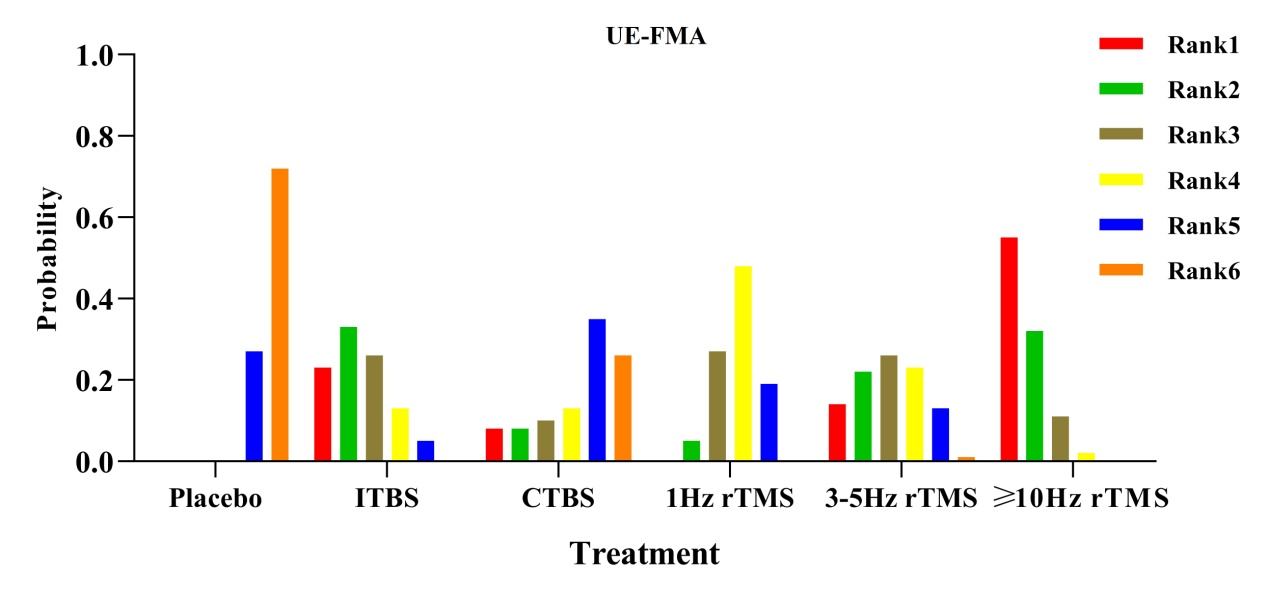


**Figure5.**Subgroup analysis of severe stroke group (UE-FMA score <33) .A:Forest plot of subgroupanalysis.B:The ranking probability of subgroup analysis.

**A**


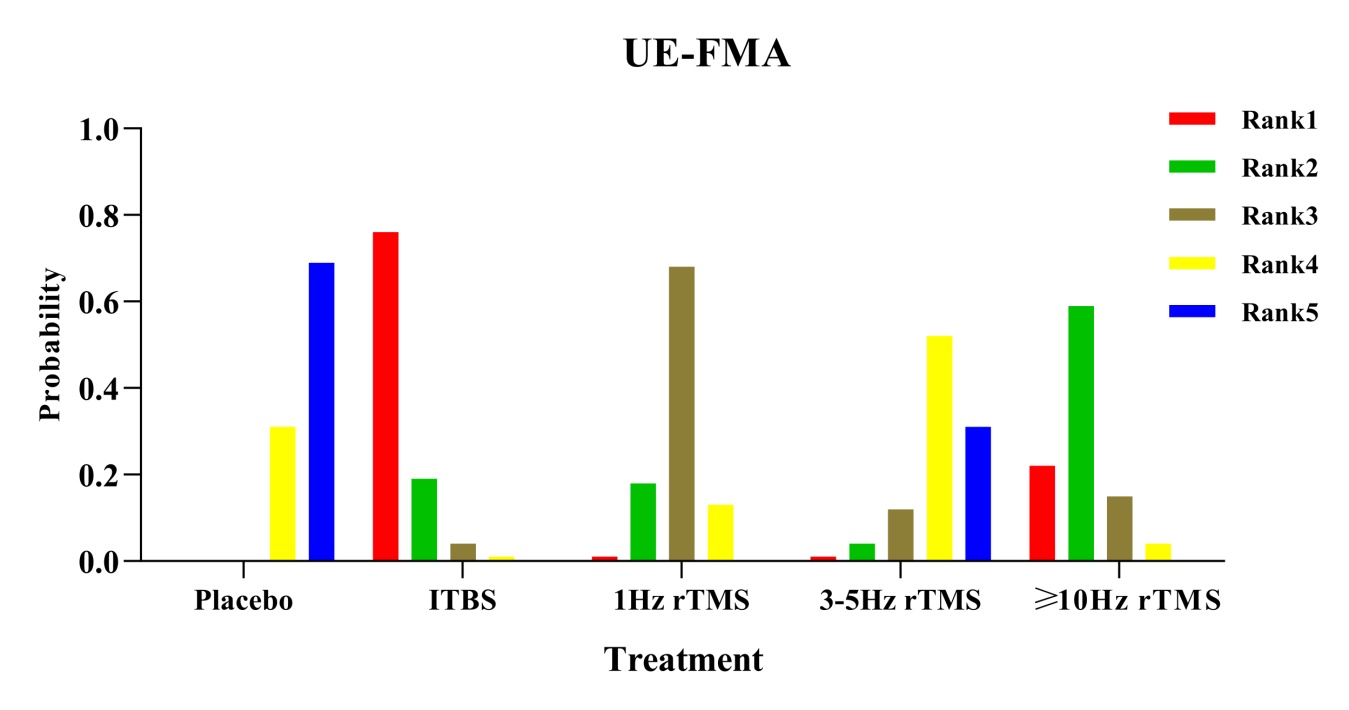
**
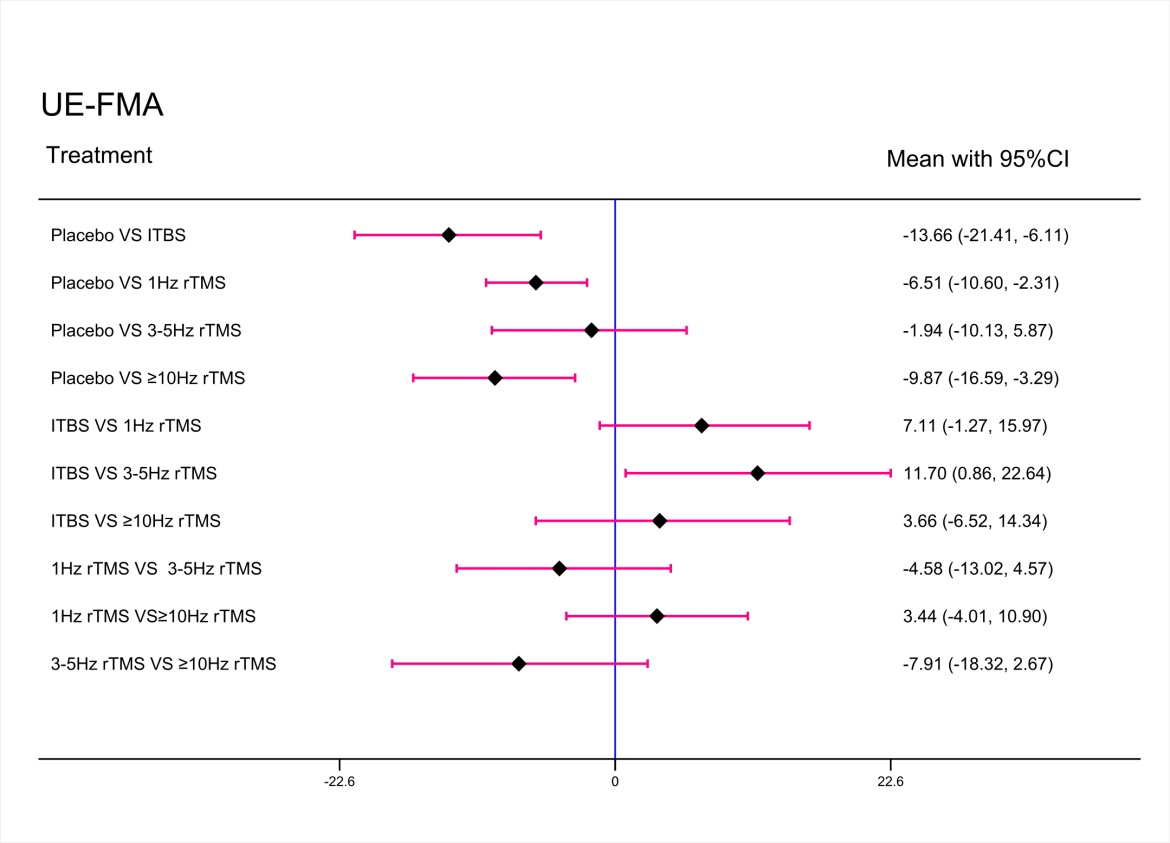
B**

**Figure6.** Subgroup analysis of acute phase and subacute phase group (<1 month).A: Forest plot of subgroup analysis.B:The ranking probability of subgroup analysis.

**A**


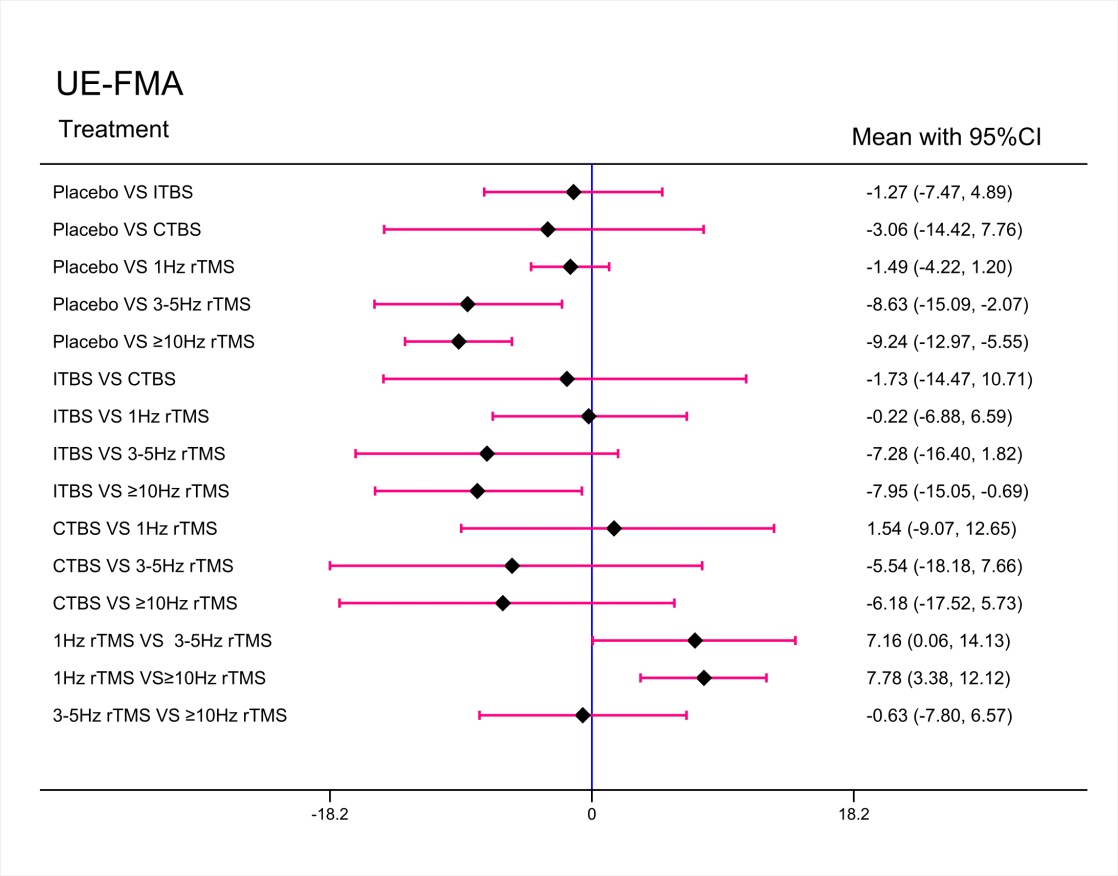


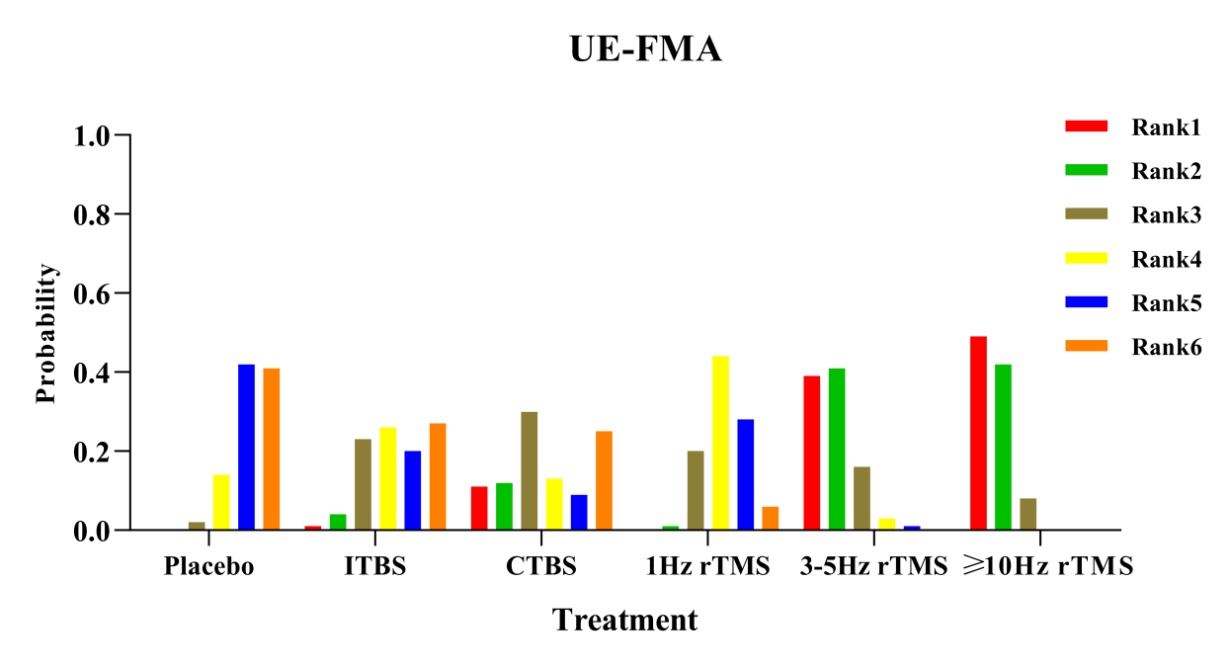
**B**

**Figure7.** Subgroup analysis of convalescent phase group (>1 month). A: Forest plot of subgroup analysis.B:The ranking probability of subgroup analysis.

**References**

1. Ackerley SJ, Byblow WD, Barber PA, MacDonald H, McIntyre-Robinson A, Stinear CM. Primed Physical Therapy Enhances Recovery of Upper Limb Function in Chronic Stroke Patients. *Neurorehabil Neural Repair*2016; **30**:339-48.

2. Chen YJ, Huang YZ, Chen CY*, et al.* Intermittent theta burst stimulation enhances upper limb motor function in patients with chronic stroke: a pilot randomized controlled trial. *BMC Neurol*2019; **19**:69.

3. Di Lazzaro V, Capone F, Di Pino G*, et al.* Combining Robotic Training and Non-Invasive Brain Stimulation in Severe Upper Limb-Impaired Chronic Stroke Patients. *Front Neurosci*2016; **10**:88.

4. Hsu YF, Huang YZ, Lin YY*, et al.* Intermittent theta burst stimulation over ipsilesional primary motor cortex of subacute ischemic stroke patients: a pilot study. *Brain Stimul*2013; **6**:166-74.

5. Khan F, Rathore C, Kate M*, et al.* The comparative efficacy of theta burst stimulation or functional electrical stimulation when combined with physical therapy after stroke: a randomized controlled trial. *Clin Rehabil*2019; **33**:693-703.

6. Koch G, Bonnì S, Casula EP*, et al.* Effect of Cerebellar Stimulation on Gait and Balance Recovery in Patients With Hemiparetic Stroke: A Randomized Clinical Trial. *JAMA Neurol*2019; **76**:170-178.

7. Kondo T, Yamada N, Momosaki R, Shimizu M, Abo M. Comparison of the Effect of Low-Frequency Repetitive Transcranial Magnetic Stimulation with That of Theta Burst Stimulation on Upper Limb Motor Function in Poststroke Patients. *Biomed Res Int*2017; **2017**:4269435.

8. Nicolo P, Magnin C, Pedrazzini E*, et al.* Comparison of Neuroplastic Responses to Cathodal Transcranial Direct Current Stimulation and Continuous Theta Burst Stimulation in Subacute Stroke. *Arch Phys Med Rehabil*2018; **99**:862-872.e1.

9. Watanabe K, Kudo Y, Sugawara E*, et al.* Comparative study of ipsilesional and contralesional repetitive transcranial magnetic stimulations for acute infarction. *J Neurol Sci*2018; **384**:10-14.

10. Tang XW HRP, Zhu YL FSJ, Wu JF YKW, Xie HY WY. The effect of intermittent theta burst stimulation on motor dysfunction after stroke. *Chinese Journal of Rehabilitation Medicine*2018; **33**:1410-1415.

11. Jiang C. Research on effect of intermittent Theta burst stimulation and 1Hz repetitive transcranial magnetic stimulation on recovering upper limb function of stroke patients.

12. Xiang WP, Wang B J, Xue H*, et al.* Effect of repetitive transcranial magnetic stimulation on motor function recovery in patients with acute ischemic stroke. *International Journal of Cerebrovascular Diseases*2017; **25**:218-222.

13. Sung WH, Wang CP, Chou CL, Chen YC, Chang YC, Tsai PY. Efficacy of coupling inhibitory and facilitatory repetitive transcranial magnetic stimulation to enhance motor recovery in hemiplegic stroke patients. *Stroke*2013; **44**:1375-82.

14. Miao YJ.Gan Zh SXS, Kan XL MJ, Lu X HYF. The effect of intermittent θburst stimulation on sEMG and function of biceps brachii and triceps in patients with cerebral infarction. *Chinese Journal of Rehabilitation Medicine*2020; **35**:440-446.

15. Guo Z, Jin Y, Peng H*, et al.* Ipsilesional High Frequency Repetitive Transcranial Magnetic Stimulation Add-On Therapy Improved Diffusion Parameters of Stroke Patients with Motor Dysfunction: A Preliminary DTI Study. *Neural Plast*2016; **2016**:6238575.

16. Hosomi K, Morris S, Sakamoto T*, et al.* Daily Repetitive Transcranial Magnetic Stimulation for Poststroke Upper Limb Paresis in the Subacute Period. *J Stroke Cerebrovasc Dis*2016; **25**:1655-1664.

17. Mahdy Ibrahim E, Ahmed Zaki M, Gaber Mahmoud Gabr M. EFFECT OF HIGH FREQUENCY REPETITIVE TRANSCRANIAL MAGNETIC STIMULATION OF THE CONTRALESIONAL MOTOR CORTEX ON RECOVERY FROM POST-STROKE SEVERE MOTOR IMPAIRMENT. *Al-Azhar Medical Journal*2020; **49**:651-666.

18. Ke J, Zou X, Huang M, Huang Q, Li H, Zhou X. High-frequency rTMS with two different inter-train intervals improves upper limb motor function at the early stage of stroke. *J Int Med Res*2020; **48**:0300060520928737.

19. Chang WH, Kim YH, Bang OY, Kim ST, Park YH, Lee PK. Long-term effects of rTMS on motor recovery in patients after subacute stroke. *J Rehabil Med*2010; **42**:758-64.

20. Wang Q, Zhang D, Zhao YY, Hai H, Ma YW. Effects of high-frequency repetitive transcranial magnetic stimulation over the contralesional motor cortex on motor recovery in severe hemiplegic stroke: A randomized clinical trial. *Brain Stimul*2020; **13**:979-986.

21. Chen CY YH, Hui N ZHK, Wang SY. Effects of transcranial magnetic stimulation combined with rehabilitation robot training on unilateral neglect and visual electrophysiology in patients with stroke. *Hainan Medical Journal*2020; **31**:2187-2190.

22. Chen YJ YQ, Cui W XL. The application of transcranial magnetic stimulation combined with somatosensory evoked potential and motor evoked potential in the treatment of cerebral apoplexy. *The Journal of Practical Medicine*2018; **34**:4115-4119.

23. Chen ZY GJQ, Sun YT GHL. Repetitive transcranial magnetic stimulation for upper limbs of patients with shoulder pain after stroke Motor function and daily activity ability. *Journal of Chinese Physician*2019; **21**:131-133.

24. Chen ZY GJQ, Wu YF PRX, Sun YT NWW, Gu HL. Repetitive transcranial magnetic stimulation combined with cognitive rehabilitation training for the treatment of post-stroke Observation on the curative effect of cognitive impairment. *Chinese Journal of Physical Medicine and Rehabilitation*2019 :199-201.

25. Liu JF WH, Xu HY ZMY. Effects of transcranial magnetic stimulation combined with functional training on neurological function and limb function in patients with stroke. *Hainan Medical Journal*2020; **31**:1926-1929.

26. Liu Y. _x000d__x000a_Effects of repetitive transcranial magnetic stimulation on recovery of upper limb motor _x000d__x000a_function in stroke patients. *Journal of Changchun University of Chinese Medicine*2020; **36**:965-967.

27. Liu Y ZCL, Qin Y. Therapeutic effect of high frequency rTMS on upper limb spasticity after stroke. *Chinese and Foreign MedicaI Research*2019; **17**:11-13.

28. Pan RR ZYH, Zhou LS. Observation on the efficacy of repetitive transcranial magnetic stimulation in the treatment of complex regional pain syndrome after stroke. *Chinese Journal of Physical Medicine and Rehabilitation*2018; **40**:671-673.

29. Su CX YR, Gong ZK WSY, Wang M. Clinical observation of abdominal acupuncture combined with repetitive transcranial magnetic stimulation in the treatment of hemiplegic shoulder pain. *_x000d__x000a_China Medical Herald*2018; **15**:117-120+128.

30. Sun SJ ZRX, Zhao JR ZXJ, Cheng XY. Effects of high frequency repetitive transcranial magnetic stimulation on anxiety and neural function rehabilitation of patients with acute cerebral infarction. *Heibei Medical Journal*2018; **40**:421-424.

31. Xiao CL PCH, Chen Y HN, Huang SK LQ, Fu Z, Ou XJ LLJ. Effects of High-frequency Repetitive Transcranial Magnetic Stimulation in Different Frequencies on Upper Limb Function after Ischemic Stroke. *Chinese Journal of Rehabilitation Theory and Practice*2019; **25**:557-563.

32. Xiao CL PCH, Chen Y YZM, Fu Z JRR, Lin WQ LLJ. Effect of High-frequency Repetitive Transcranial Magnetic Stimulation onHand Function in Patients after Stroke. *Chinese Journal of Rehabilitation Theory and Practice*2018; **24**:179-183.

33. Yi MY LJ, Hu XQ XQL, Huang L ZSX, Ai YN. Effects of high frequence repetitive transcranial magnetic stimuIation on post—stroke cognitive impairment. *Chinese Journal of Rehabilitation Medicine*2018; **33**:763-769.

34. You Q FZY, Xie LF GLT. Clinic research of repetitive transcranial magnetic stimulation combined with rehabilitation treatment for hemiplegia fol‐ lowing cerebral infarction. *Chinese Journal of Rehabilitation*2015; **30**:164-166.

35. You GQ LHY, You GJ HZ. Randomized controll study of repetitive transcranial magnetic stimulation in improvement of upper limb motor function of subacute ischemic stroke patients. *China Medicine And Pharmacy*2017; **7**:187-189+213.

36. Zhang N LXZ, Zhang FJ. Effect of transcranial magnetic stimulation on functional recovery in patients with stroke. *_x000d__x000a_Chinese Journal of Physical Medicine and Rehabilitation*2014; **36**:689-690.

37. Liang QT ZYT, Shi XG WYX, Luo XN ZCW. Effect of high—frequency repetitive transcranial magnetic stimulation on motor and sensory function of upper limbs in ischemic stroke patients. *_x000d__x000a_Chinese Journal of Geriatric Heart Brain and Vessel Diseases*2018; **20**:1187-1190.

38. Zhang XH DJF. Observation on the efficacy of transcranial magnetic stimulation in the treatment of acute ischemic stroke. *Chinese Journal of Physical Medicine and Rehabilitation*2016; **38**:523-524.

39. Zheng J SJJ, Gu LP GXH, Li Z JL, Chen DY LY. Therapeutic effects of high frequency repetitive transcranial magnetic stimulation in treating vascular cognitive impairment after stroke bat no dementia. *Chinese Journal of Rehabilitation*2017; **32**:488-491.

40. Zhou Z SXF, Xiong L ZW, Huang HY ZP, Wu Y RJF. Effects of High-frequency Repetitive Transcranial Magnetic Stimulation to Premotor Areas on Upper Limb Motor Dysfunction after Stroke. *Chinese Journal of Rehabilitation Theory and Practice*2020; **26**:697-702.

41. Ozkeskin M, Ozturk V, Cakmur R, Kara B. Navigated Repetitive Transcranial Magnetic Stimulation or Brunnstrom Hand Manipulation: Which Treatment is More Effective in Stroke Cases. *J Neurol Sci Turk*2016; **33**:361-372.

42. Wang HB LH, Yuan H DQ, Hui N WH, Miao L MX. Effect of low -frequency repetitive transcranial magnetic stimulation combining task-oriented training on upper limb motor function recovery after stroke. *Chinese Journal of Contemporary Neurology and Neurosurgery*2017; **17**:254-260.

43. Abo M, Kakuda W, Momosaki R*, et al.* Randomized, multicenter, comparative study of NEURO versus CIMT in poststroke patients with upper limb hemiparesis: the NEURO-VERIFY Study. *Int J Stroke*2014; **9**:607-12.

44. Aşkın A, Tosun A, Demirdal ÜS. Effects of low-frequency repetitive transcranial magnetic stimulation on upper extremity motor recovery and functional outcomes in chronic stroke patients: A randomized controlled trial. *Somatosens Mot Res*2017; **34**:102-107.

45. Chervyakov AV, Chervyakov AV, Poydasheva AG*, et al.* Navigated repetitive transcranial magnetic stimulation in post-stroke rehabilitation: a randomized, double-blind, sham-controlled study. *Annals of Clinical and Experimental Neurology*2015; **9**:30.

46. Chervyakov AV, Poydasheva AG, Lyukmanov RH*, et al.* Effects of Navigated Repetitive Transcranial Magnetic Stimulation After Stroke. *J Clin Neurophysiol*2018; **35**:166-172.

47. Du J, Yang F, Hu J*, et al.* Effects of high- and low-frequency repetitive transcranial magnetic stimulation on motor recovery in early stroke patients: Evidence from a randomized controlled trial with clinical, neurophysiological and functional imaging assessments. *Neuroimage Clin*2019; **21**:101620.

48. Forogh B, Ahadi T, Nazari M*, et al.* The Effect of Repetitive Transcranial Magnetic Stimulation on Postural Stability After Acute Stroke: A Clinical Trial. *Basic Clin Neurosci*2017; **8**:405-411.

49. Galvão SCB, Dos Santos RBC, Dos Santos PB, Cabral ME, Monte-Silva K. Efficacy of coupling repetitive transcranial magnetic stimulation and physical therapy to reduce upper-limb spasticity in patients with stroke: a randomized controlled trial. *Arch Phys Med Rehabil*2014; **95**:222.

50. Harvey RL, Edwards D, Dunning K*, et al.* Randomized Sham-Controlled Trial of Navigated Repetitive Transcranial Magnetic Stimulation for Motor Recovery in Stroke. *Stroke*2018; **49**:2138-2146.

51. Li J, Zhang XW, Zuo ZT*, et al.* Cerebral Functional Reorganization in Ischemic Stroke after Repetitive Transcranial Magnetic Stimulation: An fMRI Study. *CNS Neurosci Ther*2016; **22**:952-960.

52. Khedr EM, Abdel-Fadeil MR, Farghali A, Qaid M. Role of 1 and 3 Hz repetitive transcranial magnetic stimulation on motor function recovery after acute ischaemic stroke. *Eur J Neurol*2009; **16**:1323-30.

53. Kim BR, Kim DY, Chun MH, Yi JH, Kwon JS. Effect of repetitive transcranial magnetic stimulation on cognition and mood in stroke patients: a double-blind, sham-controlled trial. *Am J Phys Med Rehabil*2010; **89**:362-8.

54. Li J, Meng XM, Li RY, Zhang R, Zhang Z, Du YF. Effects of different frequencies of repetitive transcranial magnetic stimulation on the recovery of upper limb motor dysfunction in patients with subacute cerebral infarction. *Neural Regen Res*2016; **11**:1584-1590.

55. Long H, Wang H, Zhao C*, et al.* Effects of combining high- and low-frequency repetitive transcranial magnetic stimulation on upper limb hemiparesis in the early phase of stroke. *Restor Neurol Neurosci*2018; **36**:21-30.

56. Ludemann-Podubecka J, Bosl K, Theilig S, Wiederer R, Nowak DA. The Effectiveness of 1Hz rTMS Over the Primary Motor Area of the Unaffected Hemisphere to Improve Hand Function After Stroke Depends on Hemispheric Dominance. *Brain Stimul*2015; **8**:823-830.

57. Matsuura A, Onoda K, Oguro H, Yamaguchi S. Magnetic stimulation and movement-related cortical activity for acute stroke with hemiparesis. *Eur J Neurol*2015; **22**:1526-32.

58. Meng ZY, Song WQ. Low frequency repetitive transcranial magnetic stimulation improves motor dysfunction after cerebral infarction. *Neural Regen Res*2017; **12**:610-613.

59. Niimi M, Ishima T, Hashimoto K, Hara T, Yamada N, Abo M. Effect of repetitive transcranial magnetic stimulation on the kynurenine pathway in stroke patients. *Neuroreport*2020; **31**:629-636.

60. Rose DK, Patten C, McGuirk TE, Lu X, Triggs WJ. Does inhibitory repetitive transcranial magnetic stimulation augment functional task practice to improve arm recovery in chronic stroke. *Stroke Res Treat*2014; **2014**:305236.

61. Zhao N, Zhang J, Qiu M*, et al.* Scalp acupuncture plus low-frequency rTMS promotes repair of brain white matter tracts in stroke patients: A DTI study. *J Integr Neurosci*2018; **17**:125.

62. Seniów J, Bilik M, Leśniak M, Waldowski K, Iwański S, Członkowska A. Transcranial magnetic stimulation combined with physiotherapy in rehabilitation of poststroke hemiparesis: a randomized, double-blind, placebo-controlled study. *Neurorehabil Neural Repair*2012; **26**:1072-9.

63. Sharma H, Vishnu VY, Kumar N*, et al.* Efficacy of Low-Frequency Repetitive Transcranial Magnetic Stimulation in Ischemic Stroke: A Double-Blind Randomized Controlled Trial. *Arch Rehabil Res Clin Transl*2020; **2**:100039.

64. Tosun A, Türe S, Askin A*, et al.* Effects of low-frequency repetitive transcranial magnetic stimulation and neuromuscular electrical stimulation on upper extremity motor recovery in the early period after stroke: a preliminary study. *Top Stroke Rehabil*2017; **24**:361-367.

65. Motamed Vaziri P, Bahrpeyma F, Firoozabadi M*, et al.* Low frequency repetitive transcranial magnetic stimulation to improve motor function and grip force of upper limbs of patients with hemiplegia. *Iran Red Crescent Med J*2014; **16**:e13579.

66. Kim WS, Kwon BS, Seo HG, Park J, Paik NJ. Low-Frequency Repetitive Transcranial Magnetic Stimulation Over Contralesional Motor Cortex for Motor Recovery in Subacute Ischemic Stroke: A Randomized Sham-Controlled Trial. *Neurorehabil Neural Repair*2020; **34**:856-867.

67. Yang NY, Fong KN, Li-Tsang CW, Zhou D. Effects of repetitive transcranial magnetic stimulation combined with sensory cueing on unilateral neglect in subacute patients with right hemispheric stroke: a randomized controlled study. *Clin Rehabil*2017; **31**:1154-1163.

68. Zheng C, Liao W, Xia W. Effect of combined low-frequency repetitive transcranial magnetic stimulation and virtual reality training on upper limb function in subacute stroke: a double-blind randomized controlled trail. *Journal of Huazhong University of Science and Technology [Medical Sciences]*2015; **35**:248.

69. Guan Y, Li J, Zhang X*, et al.* Effectiveness of repetitive transcranial magnetic stimulation (rTMS) after acute stroke: A one‐year longitudinal randomized trial. *CNS Neurosci Ther*2017; **23**:940.

70. Cha HG. The Effect of 1 Hz Repetitive Transcranial Magnetic Stimulation Combined with Task-oriented Training on Upper Limb Function and Hemineglect in Stroke Patients. *JOURNAL OF MAGNETICS*2017; **22**:514-518.

71. Du J, Tian L, Liu W*, et al.* Effects of repetitive transcranial magnetic stimulation on motor recovery and motor cortex excitability in patients with stroke: a randomized controlled trial. *Eur J Neurol*2016; **23**:1666-1672.

72. Kim JH, Han JY, Song MK, Park GC, Lee JS. Synergistic Effects of Scalp Acupuncture and Repetitive Transcranial Magnetic Stimulation on Cerebral Infarction: A Randomized Controlled Pilot Trial. *Brain Sci*2020; **10**.

73. Zhao LN ZZQZLX, Liang WD. Effect of 1 Hz Repetitive Transcranial Magnetic Stimulation on Upper Limb Motor Function after Stroke. *_x000d__x000a_Chinese Journal of Rehabilitation Theory and Practice*2015; **21**:216-219.

74. Fu K ST. Effect of rTMS combined with forced exercise on upper limb motor function in stroke patients with stroke. *Contemp Med*2013; **19**:142-143.

75. Ge YC. Effect of low frequence repetitive transcranial magnetic stimulation on the recovery of motor function in the patients with acute stroke.

76. Wang H. Effect of low frequency repetitive transcranial magnetic stimulation on cognitive function and activities of daily living in patients with stroke. *International Medicine and Health Guidance News*2018; **24**:308-309+312.

77. Liao GH. Effect of low frequency repetitive transcranial magnetic stimulation on functional rehabilitation of convalescent stroke patients. *Modern Diagnosis and Treatment*2017; **28**:4594-4596.

78. Liu Y WXY, Zhang CL HDE, Guo XP XH, Wu HB CJR, Chen JJ QY. Effects of Low-frequency Repetitive Transcranial Magnetic Stimulation on Upper Limb Spasticity after Stroke: A Task-state Functional MagneticResonance Study. *Chinese Journal of Rehabilitation Theory and Practice*2018; **24**:828-833.

79. Liu SH LZGH, Guan CX LL, Hao DJ LY. Clinical research on the influence of low—frequency repetitive transcmnial magnetic stimulation on spasticity and motor function of patients after stroke. *_x000d__x000a_Chinese Journal of Rehabilitation Medicine*2019; **34**:1328-1332.

80. Zhang JJ JJY, Tian T JNN. Effect of low frequency repetitive transcranial magnetic stimulation combined with upper limb rehabilitation robot on upper limb motor function in patients with stroke. *Journal of Nantong University (Medical Sciences )*2019; **39**:322-324.

81. Yang YF LJH, Wu BH GJW. Clinical study on low-frequency rTMS combined with fasudil in the treatment of acute cerebral infarction. *_x000d__x000a_Journal of Hainan Medical University*2019; **25**:1787-1791+1796.

82. Ren XS WUK. Effect of low frequency repetitive transcranial magnetic stimulation combined with occupational therapy on rehabilitation of upper limb function after stroke. *_x000d__x000a_Journal of Medical Sciencein Central Sauh China*2018; **46**:132-135.

83. Lu C JDD, Fu XQ WZL, Shi AM. Effect of low frequency repetitive transcranial magnetic stimulation combined with group rehabilitation therapy on upper limb and hand functions in hemiplegic patients after stroke. *Chinese Journal of Rehabilitation*2018; **33**:369-372.

84. Zhang Y LWJ, Hao CZ. Low Repetitive Transcranial Magnetic Stimulation Combined with Occupational Therapy Improves Upper Iimb Function Post-stroke. *Chinese Journal of Rehabilitation*2019; **34**:142-145.

85. Sun W ZCG, Mou X LW, Yuan H. Clinical study of low frequency repetitive transcranial magnetic stimulation in the treatment of upper limb spasm in patients with stroke. *Chinese Journal of Rehabilitation*2017; **32**:102-105.

86. Lin W. Clinical Observation of Transcranial Magnetic Stimulation Combined with Jin's Needles in Treating Upper Limb Dysfunction in Stroke Hemiplegia Patients. *Clinical Medicine&Engineering*2019; **26**:1017-1018.

87. Cui HC ZHW, Zhang M CW. Effect of virtual reality alliance frequency combined with repetitive Transcranial magnetic stimulation on the upper limb dysfunction after stroke. *Journal of Clinical and Pathological Research*2017; **37**:2439-2444.

88. Li Q CRD, Weng WS YXM. Evaluation on the efficacy of repetitive facilitative exercise combined with low—frequency repetitive transcranial magnetic stimulation for post—stroke upper extremity dysfunction. *China Modern Doctor*2018; **56**:1-5+10.

89. Lin Min-ting LM, Yang Xuan ZX. Influence of repetitive transcranial magnetic stimulation on central motor conduction function in patients with stroke. *CHINA MODERN MEDCINE*2017; **24**:45-47.

90. Wang HL ZZH, Li G. Application of transcranial magnetic stimulation in functional rehabilitation of hemiplegic limbs in patients with stroke. *The Journal of Practical Medicine*2010; **26**:2336-2338.

91. Xiang WP, Wang BJ, Xue H, An X, Zhang J, Pang JX. The clinical observation of repetitive transcranial magnetic stimulation combined with rehabilitation for stroke patients with hemiplegia shoulder pain and upper limb movement. *Beijing Medical Journal*2015; **37**:445-447.

92. Zhao N LH, Yang WZ WHG, Wu J LP. Impacts of scalp acupuncture and repetitive transcranial magnetic stimulation on neural electrophysiological indicators in the patients of hemiplegia. *World Journal of Integrated traditional and Western Medicine*2017; **12**:1432-1435.

93. Zhao XL LTL, Zhou YX ZLX. The effect of repetitive transcranial magnetic stimulation on dyskinesia in stroke patient. *Chinese Journal of Rehabilitation Medicine*2018; **33**:800-805.

94. Zhou WN ZL, Li RD XKQ, Wu CJ. Effect of repetitive transcranial magnetic stimulation combined with exercise therapy on motor function in stroke patients with hemiplegia. *Rehabilitation Medicine*2020; **30**:235-239.

95. Xue H WBJ, Liu GR LYC, Xiang WP ZJ, Xu Y. The clinical study of high and low—frequency repetitive transcranial magnetic stimulation on motor function recovery in patients with acute ischemic stroke. *Chinese Journal of Rehabilitation Medicine*2013; **28**:1030-1034.
